# Finite‐volume scheme for a degenerate cross‐diffusion model motivated from ion transport

**DOI:** 10.1002/num.22313

**Published:** 2018-08-20

**Authors:** Clément Cancès, Claire Chainais‐Hillairet, Anita Gerstenmayer, Ansgar Jüngel

**Affiliations:** ^1^ Inria, Université de Lille CNRS, UMR 8524—Laboratoire Paul Painlevé Lille France; ^2^ Institute for Analysis and Scientific Computing Vienna University of Technology Wien Austria

**Keywords:** calcium‐selective ion channel, convergence of the scheme, entropy method, existence of discrete solutions, finite‐volume method, gradient flow, ion transport

## Abstract

An implicit Euler finite‐volume scheme for a degenerate cross‐diffusion system describing the ion transport through biological membranes is proposed. The strongly coupled equations for the ion concentrations include drift terms involving the electric potential, which is coupled to the concentrations through the Poisson equation. The cross‐diffusion system possesses a formal gradient‐flow structure revealing nonstandard degeneracies, which lead to considerable mathematical difficulties. The finite‐volume scheme is based on two‐point flux approximations with “double” upwind mobilities. The existence of solutions to the fully discrete scheme is proved. When the particles are not distinguishable and the dynamics is driven by cross diffusion only, it is shown that the scheme preserves the structure of the equations like nonnegativity, upper bounds, and entropy dissipation. The degeneracy is overcome by proving a new discrete Aubin–Lions lemma of “degenerate” type. Numerical simulations of a calcium‐selective ion channel in two space dimensions show that the scheme is efficient even in the general case of ion transport.

## 1 INTRODUCTION

The ion transport through biological channels plays an important role in all living organisms. On a macroscopic level, the transport can be described by nonlinear partial differential equations for the ion concentrations (or, more precisely, volume fractions) and the surrounding electric potential. A classical model for ion transport are the Poisson–Nernst–Planck equations 1, which satisfy Fick's law for the fluxes. However, this approach does not include size exclusion effects in narrow ion channels. Taking into account the finite size of the ions, one can derive from an on‐lattice model in the diffusion limit another set of differential equations with fluxes depending on the gradients of all species 2, 3. These nonlinear cross‐diffusion terms are common in multicomponent systems [4, Chap. 4].

In the general case, the evolution of the concentrations *u*_*i*_ and fluxes ℱ_*i*_ of the *i*th ion species is governed by the equations

(1) $$\partial_{t}u_{i}+\mathrm{div}\;{ℱ}_{i}=0,\;{ℱ}_{i}=-D_{i}\left(u_{0}\nabla u_{i}-u_{i}\nabla u_{0}+u_{0}u_{i}\beta z_{i}\nabla \Phi \right)\;\text{in}\;\Omega ,\;t>0,$$

for *i* = 1, …, *n*, where $u_{0}=1-\sum_{i=1}^{n}u_{i}$ is the concentration (volume fraction) of the electro‐neutral solvent, *D*_*i*_ > 0 is a diffusion coefficient, *β* > 0 is the (scaled) inverse thermal voltage, and *z*_*i*_ ∈ ℝ the charge of the *i*th species. Observe that we assumed Einstein's relation which says that the quotient of the diffusion and mobility coefficients is constant, and we call this constant 1/*β*. The electric potential is determined by the Poisson equation

(2) $$-\lambda^{2}\Delta \Phi =\sum_{i=1}^{n}z_{i}u_{i}+f\;\text{in}\;\Omega ,$$

where *λ* ^2^ is the (scaled) permittivity constant and *f* = *f*(*x*) is a permanent background charge density. Equations (1) and (2) are solved in a bounded domain Ω ⊂ ℝ^*d*^.

In order to match experimental conditions, the boundary *∂*Ω is supposed to consist of two parts, the insulating part Γ_*N*_, on which no‐flux boundary conditions are prescribed, and the union Γ_*D*_ of boundary contacts with external reservoirs, on which the concentrations are fixed. The electric potential is prescribed at the electrodes on Γ_*D*_. This leads to the mixed Dirichlet–Neumann boundary conditions

(3) $$\begin{array}{cc} {ℱ}_{i}\cdot \nu & =0\;\mathrm{on}\;\Gamma_{N},\;u_{i}={\bar{u}}_{i}\;\mathrm{on}\;\Gamma_{D},\;i=1,\ldots ,n, \end{array}$$

(4) $$\begin{array}{cc} \nabla \Phi \cdot \nu & =0\;\mathrm{on}\;\Gamma_{N},\;\Phi =\bar{\Phi }\;\mathrm{on}\;\Gamma_{D}, \end{array}$$

where the boundary data ${\left({\bar{u}}_{i}\right)}_{1\leq i\leq n}$ and $\bar{\Phi }$ can be defined on the whole domain Ω. Finally, we prescribe the initial conditions

(5) $$u_{i}\left(\cdot ,0\right)=u_{i}^{I}\;\text{in}\;\Omega ,\;i=1,\ldots ,n.$$

The main mathematical difficulties of Equations (1) are the strong coupling and the fact that the diffusion matrix (*A*_*ij*_(*u*)), defined by *A*_*ij*_(*u*) = *D*_*i*_ *u*_*i*_ for *i* ≠ *j* and *A*_*ii*_(*u*) = *D*_*i*_(*u*_0_ + *u*_*i*_) is not symmetric and not positive definite. It was shown in Burger and coworkers 2, 5 that system (1) possesses a formal gradient‐flow structure. This means that there exists a (relative) entropy functional *H*[*u*] = ∫_Ω_*h*(*u*)*dx* with the entropy density

$$h\left(u\right)=\sum_{i=0}^{n}\int_{{\bar{u}}_{i}}^{u_{i}}\log\frac{s}{{\bar{u}}_{i}}\mathrm{ds}+\frac{{\beta \lambda }^{2}}{2}{\left|\nabla \left(\Phi -\bar{\Phi }\right)\right|}^{2},$$

where *u* = (*u*_1_, …, *u*_*n*_) and $u_{0}=1-\sum_{i=1}^{n}u_{i}$, such that (1) can be formally written as

$$\partial_{t}u_{i}=\mathrm{div}\left(\sum_{j=1}^{n}B_{\mathrm{ij}}\nabla w_{j}\right),$$

where *B*_*ii*_ = *D*_*i*_ *u*_0_ *u*_*i*_, *B*_*ij*_ = 0 for *i* ≠ *j* provide a diagonal positive definite matrix, and *w*_*j*_ are the entropy variables, defined by

$$\begin{array}{l} \frac{\partial h}{\partial u_{i}}=w_{i}-{\bar{w}}_{i},\;\text{where}\\ w_{i}=\log\frac{u_{i}}{u_{0}}+\beta z_{i}\Phi ,\;{\bar{w}}_{i}=\log\frac{{\bar{u}}_{i}}{{\bar{u}}_{0}}+\beta z_{i}\bar{\Phi },\;i=1,\ldots ,n. \end{array}$$

We refer to [6, Lem. 7] for the computation of *∂h*/*∂u*_*i*_.

The entropy structure of (1) is useful for two reasons. First, it leads to *L*^∞^ bounds for the concentrations. Indeed, the transformation (*u*, Φ) ↦ *w* to entropy variables can be inverted, giving *u* = *u*(*w*, Φ) with

$$u_{i}\left(w,\Phi \right)=\frac{\exp\left(w_{i}-\beta z_{i}\Phi \right)}{1+\sum_{j=1}^{n}\exp\left(w_{j}-\beta z_{j}\Phi \right)},\;i=1,\ldots ,n.$$

Then *u*_*i*_ is positive and bounded from above, that is,

(6) $$u\in 𝒟=\left\{u\in {\left(0,,,1\right)}^{n}:\sum_{i=1}^{n}u_{i}<1\right\}.$$

This yields *L*^∞^ bounds without the use of a maximum principle. Second, the entropy structure leads to gradient estimates via the entropy inequality

$$\frac{\mathrm{dH}}{\mathrm{dt}}+\frac{1}{2}\int_{\Omega }\sum_{i=1}^{n}D_{i}u_{0}u_{i}{\left|\nabla w_{i}\right|}^{2}\mathrm{dx}\leq C,$$

where the constant *C* > 0 depends on the Dirichlet boundary data. Because of

(7) $$\sum_{i=1}^{n}u_{0}u_{i}{\left|\nabla \log\frac{u_{i}}{u_{0}}\right|}^{2}=4\sum_{i=1}^{n}u_{0}{\left|\nabla u_{i}^{1/2}\right|}^{2}+4{\left|\nabla u_{0}^{1/2}\right|}^{2}+{\left|\nabla u_{0}\right|}^{2},$$

we achieve gradient estimates for $u_{0}^{1/2}u_{i}$ and $u_{0}^{1/2}$. Since *u*_0_ may vanish locally, this does not give gradient bounds for *u*_*i*_, which expresses the degenerate nature of the cross‐diffusion system. As a consequence, the flux has to be formulated in the terms of gradients of $u_{0}^{1/2}u_{i}$ and $u_{0}^{1/2}$ only, namely

(8) $${ℱ}_{i}=-D_{i}\left(u_{0}^{1/2}\nabla \left(u_{0}^{1/2}u_{i}\right)-3u_{0}^{1/2}u_{i}\nabla u_{0}^{1/2}+u_{0}u_{i}\beta z_{i}\nabla \Phi \right).$$

The challenge is to derive a discrete version of this formulation. It turns out that (24) below is the right formulation in our context (assuming vanishing drift parts).

Our aim is to design a numerical approximation of (1) which preserves the structural properties of the continuous equations under simplifying assumptions (the coefficients *D*_*i*_ are the same and the drift term vanishes). This suggests to use the entropy variables as the unknowns, as it was done in our previous work 6 with simulations in one space dimension. Unfortunately, we have not been able to perform a numerical convergence analysis with these variables. The reason is that we need discrete chain rules in order to formulate (7) on the discrete level and these discrete chain rules seem to be not easily available. Therefore, we use the original variables *u*_*i*_ for the numerical discretization.

We propose a backward Euler scheme in time and a finite‐volume scheme in space, based on two‐point approximations. The key observation for the numerical discretization is that the fluxes can be written on each cell in a “double” drift‐diffusion form, that is, both ℱ_*i*_ =  − *D*_*i*_(*u*_0_*u*_*i*_ − *u*_*i*_*V*_*i*_) and *V*_*i*_ = ∇*u*_0_ − *βz*_*i*_ *u*_0_∇Φ have the structure ∇*v* + *vF*, where ∇*v* is the diffusion term and *vF* is the drift term. We discretize ℱ and *V* by using a two‐point flux aproximation with “double” upwind mobilities.

Under certain assumptions, the structure of the equations is preserved on the discrete level. Because of the drift‐diffusion structure, we are able to prove that the scheme preserves the *nonnegativity*, which follows from a discrete minimum principle argument. It is well known that the maximum priciple generally does not hold for systems of equations. Therefore, it is not a surprise that the *upper bound* comes only at a price: We need to assume that the all diffusion coefficients *D*_*i*_ are the same. Under this assumption, $u_{0}=1-\sum_{i=1}^{n}u_{i}$ solves a drift‐diffusion equation for which the (discrete) maximum principle can be applied. In order to prove that the scheme satisfies an *entropy‐dissipation inequality* and also to complete the convergence analysis of the scheme successfully, we need a stronger additional assumption: We assume that the drift terms, and therefore the coupling with the Poisson equation, can be neglected. This means that our main results are obtained for a simplified degenerate cross‐diffusion system, no more corresponding to the initial ion transport model but still of mathematical interest. Nevertheless, the scheme we propose can be applied to the full ion transport model, and this is done in the last section of this paper.

Our analytical results are stated and proved for no‐flux boundary conditions on *∂*Ω. Mixed Dirichlet–Neumann boundary conditions could be prescribed as well, but the proofs would become even more technical. The main results are as follows.

- If *D*_*i*_ = *D* for all *i*, we prove the existence of solutions to the fully discrete numerical scheme (theorem 1). If additionally the drift part vanishes, the solution is unique. The existence proof uses a topological degree argument in finite space dimensions, while the uniqueness proof is based on the entropy method of Gajewski 7, recently extended to cross‐diffusion systems 6, 8.
- If *D*_*i*_ = *D* for all *i*, the scheme preserves the nonnegativity and upper bound for the concentrations. If additionally the drift part vanishes, convexity arguments show that the discrete entropy is dissipated with a discrete entropy production analogous to (7) (theorem 2). The assumption on vanishing drift terms is needed, since a discrete version of the sum $\sum_{i=1}^{n}u_{i}$ has to be controlled from below; see the discussion after theorem 2.
- If *D*_*i*_ = *D* for all *i* and the drift part vanishes, the discrete solution converges to a continuous solution to (1) as the mesh size tends to zero (theorem 3). The proof is based on a priori estimates obtained from the discrete entropy inequality. The compactness is derived from a new discrete Aubin–Lions lemma, which takes into account the nonstandard degeneracy of the equations; see lemma 10 in the appendix.
- Numerical experiments for a calcium‐selective ion channel in two space dimensions show the dynamical behavior of the solutions and their large‐time asymptotics to the equilibrium. The tests indicate that the order of convergence in the *L* ^1^ norm is one.

In the literature, there exist some results on finite‐volume schemes for cross‐diffusion systems. An upwind two‐point flux approximation similar to our discretization was recently used in Ait Hammou Oulhaj 9 for a seawater intrusion cross‐diffusion model. A two‐point flux approximation with a nonlinear positivity‐preserving approximation of the cross‐diffusion coefficients, modeling the segregation of a two‐species population, was suggested in Andreianov and coworkers 10, assuming positive definiteness of the diffusion matrix. The Laplacian structure of the population model (still for positive definite matrices) was exploited in Murakawa 11 to design a convergent *linear* finite‐volume scheme, which avoids fully implicit approximations. A semi‐implicit finite‐volume discretization for a biofilm model with a nonlocal time integrator was proposed in Rahman and Eberl 12. Finite‐volume schemes for cross‐diffusion systems with nonlocal (in space) terms were also analyzed; see, for instance, Anaya 13 for a food chain model and 14 for an epidemic model. Moreover, a finite‐volume scheme for a Keller–Segel system with additional cross diffusion and discrete entropy dissipation property was investigated in Bessemoulin‐Chatard and Jüngel 15. All these models, however, do not include volume filling and do not possess the degenerate structure explained before.

The paper is organized as follows. The numerical scheme and the main results are presented in Section 2. In Section 3, the existence and uniqueness of bounded discrete solutions are shown. We prove the discrete entropy inequality and further a priori estimates in Section 4, while Section 5 is concerned with the convergence of the numerical scheme. Numerical experiments are given in Section 6 in order to illustrate the order of convergence and the long time behavior of the scheme. For the compactness arguments, we need two discrete Aubin–Lions lemmas which are proved in the appendix.

## 2 NUMERICAL SCHEME AND MAIN RESULTS

### 2.1 Notations and definitions

We summarize our general hypotheses on the data:

H1 Domain: Ω ⊂ ℝ^*d*^ (*d* = 2 or *d* = 3) is an open, bounded, polygonal domain with *∂*Ω = Γ_*D*_ ∪ Γ_*N*_ ∈ *C* ^0, 1^, Γ_*D*_ ∩ Γ_*N*_ = Ø.
H2 Parameters: *T* > 0, *D*_*i*_ > 0, *β* > 0, and *z*_*i*_ ∈ ℝ, *i* = 1, …, *n*.
H3 Background charge: *f* ∈ *L*^∞^(Ω).
H4 Initial and boundary data: $u_{i}^{I}\in L^{\infty }\left(\Omega \right)$, ${\bar{u}}_{i}\in H^{1}\left(\Omega \right)$ satisfy $u_{i}^{I}\geq 0$, ${\bar{u}}_{i}\geq 0$ and $1-\sum_{i=1}^{n}u_{i}^{I}\geq 0$, $1-\sum_{i=1}^{n}{\bar{u}}_{i}\geq 0$ in Ω for *i* = 1, …, *n*, and $\bar{\Phi }\in H^{1}\left(\Omega \right)\cap L^{\infty }\left(\Omega \right)$.

For our main results, we need additional technical assumptions:

A1 *∂*Ω = Γ_*N*_, that is, we impose no‐flux boundary conditions on the whole boundary.
A2 The diffusion constants are equal, *D*_*i*_ = *D* > 0 for *i* = 1, …, *n*.
A3 The drift terms are set to zero, Φ ≡ 0.

Remark 1 (Discussion of the assumptions). Assumption (A1) is supposed for simplicity only. Mixed Dirichlet–Neumann boundary conditions can be included in the analysis (see, e.g., 6), but the proofs become even more technical. Mixed boundary conditions are chosen in the numerical experiments; therefore, the numerical scheme is defined for that case. Assumption (A2) is needed for the derivation of an upper bound for the solvent concentration. Indeed, when *D*_*i*_ = *D* for all *i*, summing (1) over *i* = 1, …, *n* gives

$$\partial_{t}u_{0}=D\;\mathrm{div}\left(\nabla u_{0}-u_{0}w\nabla \Phi \right),\;\text{where}\;w=\beta \sum_{i=1}^{n}z_{i}u_{i}.$$

On the discrete level, we replace *u*_0_ *w*∇Φ by an upwind approximation. This allows us to apply the discrete maximum principle showing that *u*_0_ ≥ 0 and hence $u=\left(u_{1},\ldots ,u_{n}\right)\in \bar{𝒟}$ with 𝒟 defined in (6). Finally, Assumption (A3) is needed to derive a discrete version of the entropy inequality. Without the drift terms, the upwinding value does not depend on the index of the species, which simplifies some expressions; see Remark 2.

For the definition of the numerical scheme for (1) and (2), we need to introduce a suitable discretization of the domain Ω and the interval (0, *T*). For simplicity, we consider a uniform time discretization with time step △*t* > 0, and we set *t* ^*k*^ = *k*△*t* for *k* = 1, …, *N*, where *T* > 0, *N* ∈ ℕ are given and △*t* = *T*/*N*. The domain Ω is discretized by a regular and admissible triangulation in the sense of [16, Defin. 9.1]. The triangulation consists of a family 𝒯 of open polygonal convex subsets of Ω (so‐called cells), a family ℰ of edges (or faces in three dimensions), and a family of points ${\left(x_{K}\right)}_{K\in 𝒯}$ associated to the cells. The admissibility assumption implies that the straight line between two centers of neighboring cells $\bar{x_{K}x_{L}}$ is orthogonal to the edge *σ* = *K*|*L* between two cells *K* and *L*. The condition is satisfied by, for instance, triangular meshes whose triangles have angles smaller than *π*/2 [16, Exam. 9.1] or Voronoi meshes [16, Exam. 9.2].

We assume that the family of edges ℰ can be split into internal and external edges ℰ = ℰ_int_ ∪ ℰ_int_ with ℰ_int_ = {*σ* ∈ ℰ : *σ* ⊂ Ω} and ℰ_ext_ = {*σ* ∈ ℰ : *σ* ⊂ ∂Ω}. Each exterior edge is assumed to be an element of either the Dirichlet or Neumann boundary, that is. ${ℰ}_{\mathrm{ext}}={ℰ}_{\mathrm{ext}}^{D}\cup {ℰ}_{\mathrm{ext}}^{N}$. For given K∈𝒯, we define the set ℰ_*K*_ of the edges of *K*, which is the union of internal edges and edges on the Dirichlet or Neumann boundary, and we set ℰ_*K*, int_ = ℰ_*K*_ ∩ ℰ_int_.

The size of the mesh is defined by $h\left(𝒯\right)=\sup\left\{\text{diam}\left(K\right):K\in 𝒯\right\}$. For *σ* ∈ ℰ_int_ with *σ* = *K*|*L*, we denote by d_*σ*_ = d(*x*_*K*_, *x*_*L*_) the Euclidean distance between *x*_*K*_ and *x*_*L*_, while for *σ* ∈ ℰ_ext_, we set d_*σ*_ = *d*(*x*_*K*_, *σ*). For a given edge *σ* ∈ ℰ, the transmissibility coefficient is defined by

(9) $$\tau_{\sigma }=\frac{m\left(\sigma \right)}{d_{\sigma }},$$

where *m*(*σ*) denotes the Lebesgue measure of *σ*.

We impose a regularity assumption on the mesh: There exists *ζ* > 0 such that for all K∈𝒯 and *σ* ∈ ℰ_*K*_, it holds that

(10) $$d\left(x_{K},\sigma \right)\geq \zeta d_{\sigma }.$$

This hypothesis is needed to apply discrete functional inequalities (see 16, 17) and a discrete compactness theorem (see 18).

It remains to introduce suitable function spaces for the numerical discretization. The space ${ℋ}_{𝒯}$ of piecewise constant functions is defined by

$${ℋ}_{𝒯}=\left\{v:\bar{\Omega }\to \mathbb{R}:\exists {\left(v_{K}\right)}_{K\in 𝒯}\subset \mathbb{R},\;v\left(x\right)=\sum_{K\in 𝒯}v_{K}1_{K}\left(x\right)\right\}.$$

The (squared) discrete *H* ^1^ norm on this space is given by

(11) $${\left\|v\right\|}_{1,𝒯}^{2}=\sum_{\sigma =K|L\in {ℰ}_{\mathrm{int}}}\tau_{\sigma }{\left(v_{K}-v_{L}\right)}^{2}+\sum_{K\in 𝒯}m\left(K\right)v_{K}^{2}.$$

The discrete *H* ^−1^ norm is the dual norm with respect to the *L* ^2^ scalar product,

(12) $${\left\|v\right\|}_{-1,𝒯}=\sup\left\{\int_{\Omega }\text{vwdx}:w\in {ℋ}_{𝒯},\;{\left\|w\right\|}_{1,𝒯}=1\right\}.$$

Then

$$\left|\int_{\Omega }\text{vwdx}\right|\leq {\left\|v\right\|}_{-1,𝒯}{\left\|w\right\|}_{1,𝒯}\;\text{for}\;v,w\in {ℋ}_{𝒯}.$$

Finally, we introduce the space ${ℋ}_{𝒯,△t}$ of piecewise constant in time functions with values in ${ℋ}_{𝒯}$,

$${ℋ}_{𝒯,△t}=\left\{v:\bar{\Omega }\times \left[0,T\right]\to \mathbb{R}:\exists {\left(v^{k}\right)}_{k=1,\ldots ,N}\subset {ℋ}_{𝒯},\;v(x,t)=\sum_{k=1}^{N}v^{k}\left(x\right)1_{\left(t^{k-1},t^{k}\right)}\left(t\right)\right\},$$

equipped with the discrete *L* ^2^(0, *T*;*H* ^1^(Ω)) norm

$${\left\|v\right\|}_{1,𝒯,△t}={\left(\sum_{k=1}^{N}△t{\left\|v^{k}\right\|}_{1,𝒯}^{2}\right)}^{1/2}.$$

For the numerical scheme, we introduce some further definitions. Let $u_{i}\in {ℋ}_{𝒯}$ with values ${\bar{u}}_{i,\sigma }$ on the Dirichlet boundary (*i* = 1, …, *n*). Then we introduce

(13) $$\begin{array}{l} \;D_{K,\sigma }\left(u_{i}\right)=u_{i,K,\sigma }-u_{i,K},\\ \text{where}\;u_{i,K,\sigma }=\left\{\begin{array}{ll} u_{i,L}\; & \;\text{for}\;\sigma \in {ℰ}_{\mathrm{int}},\;\sigma =K|L,\\ {\bar{u}}_{i,\sigma }\; & \;\text{for}\;\sigma \in {ℰ}_{\mathrm{ext},K}^{D},\\ u_{i,K} & \;\text{for}\;\sigma \in {ℰ}_{\mathrm{ext},K}^{N}, \end{array}\right.\;{\bar{u}}_{i,\sigma }=\frac{1}{m\left(\sigma \right)}\int_{\sigma }{\bar{u}}_{i}\mathrm{ds}. \end{array}$$

The numerical fluxes ℱ_*K*, *σ*_ should be consistent approximations to the exact fluxes through the edges ∫_*σ*_ℱ · *νds*. We impose the conservation of the numerical fluxes ℱ_*K*, *σ*_ + ℱ_*L*, *σ*_ = 0 for edges *σ* = *K*|*L*, requiring that they vanish on the Neumann boundary edges, ℱ_*K*, *σ*_ = 0 for $\sigma \in {ℰ}_{\mathrm{ext},K}^{N}$. Then the discrete integration‐by‐parts formula becomes for $u\in {ℋ}_{𝒯}$

$$\sum_{K\in 𝒯}\sum_{\sigma \in {ℰ}_{K}}{ℱ}_{K,\sigma }u_{K}=-\sum_{\sigma \in ℰ}{ℱ}_{K,\sigma }D_{K,\sigma }\left(u\right)+\sum_{\sigma \in {ℰ}_{\mathrm{ext}}^{D}}{ℱ}_{K,\sigma }u_{K,\sigma }.$$

When *∂*Ω = Γ_*N*_, this formula simplifies to

(14) $$\sum_{K\in 𝒯}\sum_{\sigma \in {ℰ}_{K}}{ℱ}_{K,\sigma }u_{K}=\sum_{\sigma =K|L\in {ℰ}_{\mathrm{int}}}{ℱ}_{K,\sigma }\left(u_{K}-u_{L}\right).$$

### 2.2 Numerical scheme

We need to approximate the initial, boundary, and given functions on the elements K∈𝒯 and edges *σ* ∈ ℰ:

$$\begin{array}{llll} u_{i,K}^{I} & =\frac{1}{m\left(K\right)}\int_{K}u_{i}^{I}\left(x\right)\mathrm{dx}, & f_{K} & =\frac{1}{m\left(K\right)}\int_{K}f\left(x\right)\mathrm{dx},\\ {\bar{u}}_{i,\sigma } & =\frac{1}{m\left(\sigma \right)}\int_{\sigma }{\bar{u}}_{i}\mathrm{ds}, & {\bar{\Phi }}_{\sigma } & =\frac{1}{m\left(\sigma \right)}\int_{\sigma }\bar{\Phi }\mathrm{ds}, \end{array}$$

and we set $u_{0,K}^{I}=1-\sum_{i=1}^{n}u_{i,K}^{I}$ and ${\bar{u}}_{0,\sigma }=1-\sum_{i=1}^{n}{\bar{u}}_{i,\sigma }$.

The numerical scheme is as follows. Let K∈𝒯, *k* ∈{1, …, *N*}, *i* = 1, …, *n*, and $u_{i,K}^{k-1}\geq 0$ be given. Then the values $u_{i,K}^{k}$ are determined by the implicit Euler scheme

(15) $$m\left(K\right)\frac{u_{i,K}^{k}-u_{i,K}^{k-1}}{\Delta t}+\sum_{\sigma \in {ℰ}_{K}}{ℱ}_{i,K,\sigma }^{k}=0,$$

where the fluxes ${ℱ}_{i,K,\sigma }^{k}$ are given by the upwind scheme

(16) $${ℱ}_{i,K,\sigma }^{k}=-\tau_{\sigma }D_{i}\left(u_{0,\sigma }^{k}D_{K,\sigma }\left(u_{i}^{k}\right)-u_{i,\sigma }^{k}\left(D_{K,\sigma }\left(u_{0}^{k}\right)-{\hat{u}}_{0,\sigma ,i}^{k}\beta z_{i}D_{K,\sigma }\left(\Phi^{k}\right)\right)\right),$$

where *τ*_*σ*_ is defined in (9),

(17) $$u_{0,K}^{k}=1-\sum_{i=1}^{n}u_{i,K}^{k},\;u_{0,\sigma }^{k}=\max\left\{u_{0,K}^{k},u_{0,L}^{k}\right\},$$

(18) $$u_{i,\sigma }^{k}=\left\{\begin{array}{ll} u_{i,K}^{k}\; & \text{if}\;{𝒱}_{i,K,\sigma }^{k}\geq 0,\\ u_{i,K,\sigma }^{k} & \text{if}\;{𝒱}_{i,K,\sigma }^{k}<0, \end{array}\right.,\;{\hat{u}}_{0,\sigma ,i}^{k}=\left\{\begin{array}{ll} u_{0,K}^{k}\; & \text{if}\;z_{i}D_{K,\sigma }\left(\Phi^{k}\right)\geq 0,\\ u_{0,K,\sigma }^{k} & \text{if}\;z_{i}D_{K,\sigma }\left(\Phi^{k}\right)<0, \end{array}\right.,$$

and ${𝒱}_{i,K,\sigma }^{k}$ is the “drift part” of the flux,

(19) $${𝒱}_{i,K,\sigma }^{k}=D_{K,\sigma }\left(u_{0}^{k}\right)-{\hat{u}}_{0,\sigma ,i}^{k}\beta z_{i}D_{K,\sigma }\left(\Phi^{k}\right)$$

for *i* = 1, …, *n*. Observe that we employed a double upwinding: one related to the electric potential, defining ${\hat{u}}_{0,\sigma ,i}^{k}$, and another one related to the drift part of the flux, ${𝒱}_{i,K,\sigma }^{k}$. The potential is computed via

(20) $$-\lambda^{2}\sum_{\sigma \in {ℰ}_{K}}\tau_{\sigma }D_{K,\sigma }\left(\Phi^{k}\right)=m\left(K\right)\left(\sum_{i=1}^{n}z_{i}u_{i,K}^{k}+f_{K}\right).$$

We recall that the numerical boundary conditions are given by ${\bar{u}}_{i,\sigma }$ and ${\bar{\Phi }}_{\sigma }$ for $\sigma \in {ℰ}_{\mathrm{ext}}^{D}$.

We denote by $u_{i,𝒯,△t}$, $\Phi_{𝒯,△t}$ the functions in ${ℋ}_{𝒯,△t}$ associated to the values $u_{i,K}^{k}$ and $\Phi_{K}^{k}$, respectively. Moreover, when dealing with a sequence of meshes ${\left({𝒯}_{m}\right)}_{m}$ and a sequence of time steps (△*t*_*m*_)_*m*_, we set $u_{i,m}=u_{i,{𝒯}_{m},△t_{m}}$, $\Phi_{m}=\Phi_{{𝒯}_{m},△t_{m}}$.

Remark 2 (Simplified numerical scheme)When Assumptions (A1)–(A3) hold, the numerical scheme simplifies to

(21) $$m\left(K\right)\frac{u_{i,K}^{k}-u_{i,K}^{k-1}}{△t}+\sum_{\sigma \in {ℰ}_{K,\mathrm{int}}}{ℱ}_{i,K,\sigma }^{k}=0,$$

(22) $${ℱ}_{i,K,\sigma }^{k}=-\tau_{\sigma }D\left(u_{0,\sigma }^{k}\left(u_{i,L}^{k}-u_{i,K}^{k}\right)-u_{i,\sigma }^{k}\left(u_{0,L}^{k}-u_{0,K}^{k}\right)\right),$$

where $u_{0,K}^{k}$ and $u_{0,\sigma }^{k}$ are defined in (17), and the definition of $u_{i,\sigma }^{k}$ simplifies to

$$u_{i,\sigma }^{k}=\left\{\begin{array}{ll} u_{i,K}^{k} & \;\text{if}\;u_{0,K}^{k}-u_{0,L}^{k}\leq 0,\\ u_{i,L}^{k} & \;\text{if}\;u_{0,K}^{k}-u_{0,L}^{k}>0. \end{array}\right.$$

In the definition of $u_{i,\sigma }^{k}$, the upwinding value does not depend on *i* anymore such that

(23) $$\sum_{i=0}^{n}u_{i,\sigma }^{k}=1+\max\left\{u_{0,K}^{k},u_{0,L}^{k}\right\}-\min\left\{u_{0,K}^{k},u_{0,L}^{k}\right\}=1+|u_{0,K}^{k}-u_{0,L}^{k}|.$$

This property is needed to control the sum $\sum_{i=1}^{n}u_{i,\sigma }^{k}$ from below in the proof of the discrete entropy inequality; see (35). Finally, we are able to reformulate the discrete fluxes such that we obtain a discrete version of (8) (without the drift part):

(24) $${ℱ}_{i,K,\sigma }=\tau_{\sigma }D\left\{u_{0,\sigma }^{1/2}\left(u_{0,K}^{1/2}u_{i,K}-u_{0,L}^{1/2}u_{i,L}\right)-u_{i,\sigma }\left(u_{0,K}^{1/2}-u_{0,L}^{1/2}\right)\left(u_{0,\sigma }^{1/2}+2\frac{u_{0,K}^{1/2}+u_{0,L}^{1/2}}{2}\right)\right\}.$$

This formulation is needed in the convergence analysis.

### 2.3 Main results

Since our scheme is implicit and nonlinear, the existence of an approximate solution is nontrivial. Therefore, our first result concerns the well‐posedness of the numerical scheme.

Theorem 1 (Existence and uniqueness of solutions). *Let (H1)–(H4) and (A2) hold. Then there exists a solution (u, Φ) to Scheme* (15), (16), (17), (18), (19), (20) *satisfying* $u^{k}\in \bar{𝒟}$ *and, if the initial data lie in* 𝒟, $u^{k}\in 𝒟$. *If additionally Assumptions (A1) and (A3) hold, the solution is unique*.

Assumption (A2) is needed to show that $u_{0}^{k}=1-\sum_{i=1}^{n}u_{i}^{k}$ is nonnegative. Indeed, summing (15) and (16) over *i* = 1, ….*n*, we obtain

$$m\left(K\right)\frac{u_{0,K}^{k}-u_{0,K}^{k-1}}{△t}=-\sum_{\sigma \in {ℰ}_{K}}\tau_{\sigma }\left(u_{0,\sigma }^{k}D_{K,\sigma }\left(\sum_{i=1}^{n}D_{i}u_{i}^{k}\right)-\sum_{i=1}^{n}D_{i}u_{i,\sigma }^{k}{𝒱}_{i,K,\sigma }^{k}\right).$$

Under Assumption (A2), it follows that $\sum_{i=1}^{n}D_{i}u_{i,K}^{k}=D\left(1-u_{0,K}^{k}\right)$, and we can apply the discrete minimum principle, which then implies an *L*^∞^ bound for $u_{i}^{k}$. This bound allows us to apply a topological degree argument; see 19, 20. For the uniqueness proof, we additionally need Assumption (A3), since we use the entropy method of Gajewski 7, and it seems that this method cannot be applied to cross‐diffusion systems including drift terms 8. The idea is to prove first the uniqueness of $u_{0}^{k}$, which solves a discrete nonlinear equation, and then to show the uniqueness of $u_{i}^{k}$ for *i* = 1, …, *n* by introducing a semimetric *d*(*u* ^*k*^, *v* ^*k*^) for two solutions $u^{k}=\left(u_{1}^{k},\ldots ,u_{n}^{k}\right)$ and $v^{k}=\left(v_{1}^{k},\ldots ,v_{n}^{k}\right)$ and showing that it is monotone in *k*, such that a discrete Gronwall argument implies that *u* ^*k*^ = *v* ^*k*^.

The second result shows that the scheme preserves a discrete version of the entropy inequality.

Theorem 2 (Discrete entropy inequality). *Let Assumptions (H1)–(H4) and (A1)–(A3) hold. Then the solution to Scheme* (21), (22) *constructed in theorem* 1 *satisfies the discrete entropy inequality*

(25) $$\frac{H^{k}-H^{k-1}}{\Delta t}+I^{k}\leq 0,$$

*with the discrete entropy*

(26) $$H^{k}=\sum_{K\in 𝒯}m\left(K\right)\sum_{i=0}^{n}\left(u_{i,K}^{k}\left(\log u_{i,K}^{k}-1\right)+1\right)$$

*and the discrete entropy production*

$$\begin{array}{ll} I^{k} & =D\sum_{\sigma =K|L\in {ℰ}_{\mathrm{int}}}\tau_{\sigma }\left(4\sum_{i=1}^{n}u_{0,\sigma }^{k}{\left({\left(u_{i,K}^{k}\right)}^{1/2}-{\left(u_{i,L}^{k}\right)}^{1/2}\right)}^{2}\right.\\ & \;+4{\left({\left(u_{0,K}^{k}\right)}^{1/2}-{\left(u_{0,L}^{k}\right)}^{1/2}\right)}^{2}+{\left(u_{0,K}^{k}-u_{0,L}^{k}\right)}^{2}). \end{array}$$

Assumption (A3) is required to estimate the expression $\sum_{i=1}^{n}u_{i,\sigma }^{k}$. In the continuous case, this sum equals 1 − *u*_0_. On the discrete level, this identity cannot be expected since the value of $u_{i,\sigma }^{k}$ depends on the upwinding value; see (18). If the drift part vanishes, the upwinding value does not depend on i, as mentioned in remark 2, and we can derive the estimate $\sum_{i=1}^{n}u_{i,\sigma }^{k}\geq 1-u_{0,\sigma }^{k}$; see Section 4.1. Note that the entropy production I^k^ is the discrete counterpart of (7).

The main result of this paper is the convergence of the approximate solutions to a solution to the continuous cross‐diffusion system.

Theorem 3 (Convergence of the approximate solution). *Let (H1)–(H4) and (A1)–(A3) hold and let* $\left({𝒯}_{m}\right)$ *and* (△*t*_m_) *be sequences of admissible meshes and time steps, respectively, such that* $h\left({𝒯}_{m}\right)\to 0$ *and* △*t*_*m*_ → 0 *as* *m* → ∞. *Let* (*u*_*0, m*_, …, *u*_*n, m*_) *be the solution to* (21), (22) *constructed in theorem* 1. *Then there exist functions u*_0_, *u* = (*u*_1_, …, *u*_*n*_) *satisfying* $u\left(x,t\right)\in \bar{𝒟}$,

$$\begin{array}{l} u_{0}^{1/2},\;u_{0}^{1/2}u_{i}\in L^{2}\left(0,T;H^{1}\left(\Omega \right)\right),\;i=1,\ldots ,n,\\ u_{0,m}^{1/2}\to u_{0}^{1/2},\;u_{0,m}^{1/2}u_{i,m}\to u_{0}^{1/2}u_{i}\;\text{strongly in}\;L^{2}\left(\Omega \times \left(0,T\right)\right), \end{array}$$

*where u is a weak solution to* (1), (3), (4), (5) (*with* Γ_*N*_ = ∂Ω), *that is, for all* $\phi \in C_{0}^{\infty }(\bar{\Omega }\times [0,T))$ *and i = 1, …, n,*

(27) $$\int_{0}^{T}\int_{\Omega }u_{i}\partial_{t}\text{ϕdxdt}+\int_{\Omega }u_{i}^{I}\phi \left(\cdot ,0\right)\mathrm{dx}=D\int_{0}^{T}\int_{\Omega }u_{0}^{1/2}\left(\nabla \left(u_{0}^{1/2}u_{i}\right)-3u_{i}\nabla u_{0}^{1/2}\right)\cdot \nabla \text{ϕdxdt}.$$

The compactness of the concentrations follows from the discrete gradient estimates derived from the entropy inequality (25), for which we need Assumption (A3). By the discrete Aubin–Lions lemma 21, we conclude the strong convergence of the sequence $\left(u_{0,m}^{1/2}\right)$. The difficult part is to show the strong convergence of $\left(u_{0,m}^{1/2}u_{i,m}\right)$, since there is no control on the discrete gradient of *u*_*i*, *m*_. The idea is to apply a discrete Aubin–Lions lemma of “degenerate” type, proved in lemma 10 in the appendix.

## 3 EXISTENCE AND UNIQUENESS OF APPROXIMATE SOLUTIONS

### 3.1 *L*^∞^ bounds and existence of solutions

In order to prove the existence of solutions to (15), (16), (17), (18), (19), (20), we first consider a truncated problem. This means that we truncate the expressions in (18); more precisely, we consider Scheme (15), (16), and (20) with

(28) $$\begin{array}{l} u_{0,K}^{k}=1-\sum_{i=1}^{n}{\left(u_{i,K}^{k}\right)}^{+},\;u_{0,\sigma }^{k}=\max\left\{0,u_{0,K}^{k},u_{0,K,\sigma }^{k}\right\},\\ {\hat{u}}_{0,\sigma ,i}^{k}=\left\{\begin{array}{ll} {\left(u_{0,K}^{k}\right)}^{+}\; & \text{if}\;z_{i}D_{K,\sigma }\left(\Phi^{k}\right)\geq 0,\\ {\left(u_{0,K,\sigma }^{k}\right)}^{+} & \text{if}\;z_{i}D_{K,\sigma }\left(\Phi^{k}\right)<0, \end{array}\right.\\ u_{i,\sigma }^{k}=\left\{\begin{array}{ll} {\left(u_{i,K}^{k}\right)}^{+}\; & \text{if}\;{𝒱}_{i,K,\sigma }^{k}\geq 0,\\ {\left(u_{i,K,\sigma }^{k}\right)}^{+} & \text{if}\;{𝒱}_{i,K,\sigma }^{k}<0, \end{array}\right. \end{array}$$

where *z*^+^ = max {0, *z*} for *z* ∈ ℝ and *i* = 1, …, *n*. We show that this truncation is, in fact, not needed if the initial data are nonnegative. In the following let (H1)–(H4) hold.

Lemma 4 (Nonnegativity of $u_{i}^{k}$). *Let* (*u*, Φ) *be a solution to* (15), (16), (20), and (28). *Then* $u_{i,K}^{k}\geq 0$ *for all* K∈𝒯, *k ∈{1, …, N}, and i = 1, …, n. If* $u_{i}^{I}>0$ *and* ${\bar{u}}_{i}>0$ *then also* $u_{i,K}^{k}>0$ *for all* K∈𝒯, *k ∈{1, …, N}*.

We proceed by induction. For *k* = 0, the nonnegativity holds because of our assumptions on the initial data. Assume that $u_{i,L}^{k-1}\geq 0$ for all L∈𝒯. Then let $u_{i,K}^{k}=\min\left\{u_{i,L}^{k}:L\in 𝒯\right\}$ for some K∈𝒯 and assume that $u_{i,K}^{k}<0$. The scheme writes as

(29) $$m\left(K\right)\frac{u_{i,K}^{k}-u_{i,K}^{k-1}}{△t}=\sum_{\sigma \in {ℰ}_{K}}\tau_{\sigma }D_{i}\left(u_{0,\sigma }^{k}D_{K,\sigma }\left(u_{i}^{k}\right)-u_{i,\sigma }^{k}{𝒱}_{i,K,\sigma }^{k}\right).$$

By assumption, $D_{K,\sigma }\left(u_{i}^{k}\right)\geq 0$. If ${𝒱}_{i,K\sigma }^{k}\geq 0$, we have $-u_{i,\sigma }^{k}{𝒱}_{i,K,\sigma }^{k}=-{\left(u_{i,K}\right)}^{+}{𝒱}_{i,K,\sigma }=0$ and if ${𝒱}_{i,K,\sigma }^{k}<0$, it follows that $-u_{i,\sigma }^{k}{𝒱}_{i,K,\sigma }^{k}=-{\left(u_{i,K,\sigma }^{k}\right)}^{+}{𝒱}_{i,K,\sigma }^{k}\geq 0$. Hence, the right‐hand side of (29) nonnegative. However, the left‐hand side is negative, which is a contradiction. We infer that $u_{i,K}^{k}\geq 0$ and consequently, $u_{i,L}^{k}\geq 0$ for all L∈𝒯. When the initial data are positive, similar arguments show the positivity of $u_{i,L}^{k}$ for L∈𝒯.

We are able to show the nonnegativity of $u_{0,K}^{k}=1-\sum_{i=1}^{n}u_{i,K}^{k}$ only if the diffusion coefficients are the same. The reason is that we derive an equation for $u_{0,K}^{k}$ by summing (15) for *i* = 1, …, *n*, and this gives an equation for $u_{0,K}^{k}$ only if *D*_*i*_ = *D* for all *i* = 1, …, *n*.

Lemma 5 (Nonnegativity of $u_{0}^{k}$). *Let Assumption (A2) hold and let (u, Φ) be a solution to* (15), (16), (20), *and* (28). *Then* $u_{0,K}^{k}\geq 0$ *for all* K∈𝒯, *k ∈ {1, …, N}. If* $u_{0}^{I}>0$ *and* ${\bar{u}}_{i}>0$ *then also* $u_{0,K}^{k}>0$ *for all* K∈𝒯, *k ∈ {1, …, N}*.

Again, we proceed by induction. The case *k* = 0 follows from the assumptions. Assume that $u_{0,L}^{k-1}\geq 0$ for all L∈𝒯. Then let $u_{0,K}^{k}=\min\left\{u_{0,L}^{k}:L\in 𝒯\right\}$ for some K∈𝒯 and assume that $u_{0,K}^{k}<0$. Summing Equations (15) from *i* = 1, …, *n*, we obtain

(30) $$\begin{array}{ll} m\left(K\right)\frac{u_{0,K}^{k}-u_{0,K}^{k-1}}{\Delta t} & =D\sum_{\sigma \in {ℰ}_{K}}\tau_{\sigma }\left(u_{0,\sigma }^{k}D_{K,\sigma }\left(u_{0}^{k}\right)+\sum_{i=1}^{n}u_{i,\sigma }^{k}\left(D_{K,\sigma }\left(u_{0}^{k}\right)-\beta z_{i}{\hat{u}}_{0,\sigma ,i}^{k}D_{K,\sigma }\left(\Phi^{k}\right)\right)\right)\\ & \geq -D\sum_{\sigma \in {ℰ}_{K}}\tau_{\sigma }\sum_{i=1}^{n}\beta z_{i}{\hat{u}}_{0,\sigma ,i}^{k}D_{K,\sigma }\left(\Phi^{k}\right), \end{array}$$

since $u_{0,\sigma }^{k}\geq 0$ and $u_{i,\sigma }^{k}\geq 0$ by construction and $D_{K,\sigma }\left(u_{0}^{k}\right)\geq 0$ because of the minimality property of $u_{0,K}^{k}$. The remaining expression is nonnegative:

$$-{\hat{u}}_{0,\sigma_{i}}^{k}z_{i}D_{K,\sigma }\left(\Phi^{k}\right)=\left\{\begin{array}{ll} -{\left(u_{0,K}^{k}\right)}^{+}z_{i}D_{K,\sigma }\left(\Phi^{k}\right)=0\; & \text{if}\;z_{i}D_{K,\sigma }\left(\Phi^{k}\right)\geq 0,\\ -{\left(u_{0,L}^{k}\right)}^{+}z_{i}D_{K,\sigma }\left(\Phi^{k}\right)\geq 0 & \text{if}\;z_{i}D_{K,\sigma }\left(\Phi^{k}\right)<0. \end{array}\right.$$

However, the left‐hand side of (30) is negative, by induction hypothesis, which gives a contradiction.

Lemmas 4 and 5 imply that we may remove the truncation in (28). Moreover, by definition, we have $1-\sum_{i=1}^{n}u_{i,K}^{k}=u_{0,K}^{k}\geq 0$ such that $u_{K}^{k}=\left(u_{1,K}^{k},\ldots ,u_{n,K}^{k}\right)\in \bar{𝒟}$ or, if the initial and boundary data are positive, $u_{K}^{k}\in 𝒟$.

Proposition 6 (Existence for the numerical scheme). *Let Assumption (A2) hold. Then scheme* (15), (16), (17), (18), (19), (20) *has a solution (u, Φ) which satisfies* $u_{K}^{k}\in \bar{𝒟}$ *for all* K∈𝒯 *and* *k* ∈ ℕ.

We argue by induction. For *k* = 0, we have $u_{K}^{0}\in \bar{𝒟}$ by assumption. The function Φ^0^ is uniquely determined by scheme (20), as this is a linear system of equations with positive definite matrix. Assume the existence of a solution (*u* ^*k* − 1^, Φ^*k* − 1^) with $u_{K}^{k-1}\in \bar{𝒟}$. Let *m* ∈ ℕ be the product of the number of species *n* and the number of cells K∈𝒯. For given K∈𝒯 and *i* = 1, …, *n*, we define the function *F*_*i*, *K*_ : ℝ^*m*^ × [0, 1] → ℝ by

$$\begin{array}{ll} F_{i,K}\left(u,\rho \right) & =m\left(K\right)\frac{u_{i,K}-u_{i,K}^{k-1}}{△t}\\ & \;-\rho D\sum_{\sigma \in {ℰ}_{K}}\tau_{\sigma }\left(u_{0,\sigma }D_{K,\sigma }\left(u_{i}\right)-u_{i,\sigma }\left(D_{K,\sigma }\left(u_{0}\right)-{\hat{u}}_{0,\sigma ,i}\beta z_{i}D_{K,\sigma }\left(\Phi \right)\right)\right). \end{array}$$

where *u*_0, *K*_, *u*_*i*, *σ*_, *u*_0, *σ*_, and ${\hat{u}}_{0,\sigma_{i}}$ are defined in (28), and Φ is uniquely determined by (20). Let $F={\left(F_{i,K}\right)}_{i=1,\ldots ,n,K\in 𝒯}$. Then *F* : ℝ^*m*^ × [0, 1] → ℝ^*m*^ is a continuous function. We wish to apply the fixed‐point theorem of [21, Theor. 5.1]. For this, we need to verify three assumptions:

- The function $u\mapsto F_{i,K}\left(u,0\right)=m\left(K\right)\left(u_{i,K}-u_{i,K}^{k-1}\right)/△t$ is affine.
- We have proved above that any solution to *F*(*u*, 1) = 0 satisfies u∈𝒟 or ‖*u*‖_∞_ < 2. A similar proof shows that any solution to *F*(*u*, *ρ*) = 0 with *ρ* ∈ (0, 1) satisfies ‖*u*‖_∞_ < 2, too.
- The equation *F*(*u*, 0) = 0 has the unique solution *u* = *u* ^*k* − 1^ and consequently, ‖*u*‖_∞_ = ‖*u*^*k* − 1^‖_∞_ < 2.

We infer the existence of a solution *u* ^*k*^ to *F*(*u* ^*k*^, 1) = 0 satisfying ‖*u*^*k*^‖_∞_ < 2. In fact, by lemmas 4 and 5, we find that $u^{k}\in \bar{𝒟}$. Hence, *u* ^*k*^ solves the original scheme (15), (16), (17), (18), (19), (20).

### 3.2 Uniqueness of solutions

The proof of Theorem 1 is completed when we show the uniqueness of solutions to scheme (15), (16), (17), (18), (19), (20) under the additional conditions (A1) and (A3). Recall that in this case, the scheme is given by (21), (22),

*Step 1: uniqueness for u*_*0*_. If *k* = 0, the solution is uniquely determined by the initial condition. Assume that $u_{0}^{k-1}$ is given. Thanks to Assumptions (A2) and (A3), the sum of (21) and (22) for *i* = 1, …, *n* gives an equation for $u_{0}^{k}=1-\sum_{i=1}^{n}u_{i}^{k}$ (in the following, we omit the superindices *k*):

$$\begin{array}{ll} m\left(K\right)\frac{u_{0,K}-u_{0,K}^{k-1}}{△t} & =-D\sum_{\sigma \in {ℰ}_{K,\mathrm{int}}}\tau_{\sigma }\left(u_{0,K}-u_{0,L}\right)\left(u_{0,\sigma }+\sum_{i=1}^{n}u_{i,\sigma }\right)\\ & =-D\sum_{\sigma \in {ℰ}_{K,\mathrm{int}}}\tau_{\sigma }\left(u_{0,K}-u_{0,L}\right)\left(1+|u_{0,K}-u_{0,L}|\;\right), \end{array}$$

where we used (23) in the last step.

Let *u*_0_ and *v*_0_ be two solutions to the previous equation and set *w*_0_: = *u*_0_ − *v*_0_. Then *w*_0_ solves

$$\begin{array}{ll} 0 & =m\left(K\right)\frac{w_{0,K}}{△t}+D\sum_{\sigma \in {ℰ}_{K,\mathrm{int}}}\tau_{\sigma }\left(w_{0,K}-w_{0,L}\right)\\ & \;+D\sum_{\sigma \in {ℰ}_{K,\mathrm{int}}}\tau_{\sigma }\left(\left(u_{0,K}-u_{0,L}\right)|u_{0,K}-u_{0,L}|-\left(v_{0,K}-v_{0,L}\right)|v_{0,K}-v_{0,L}|\;\right). \end{array}$$

We multiply this equation by *w*_0, *K*_/*D*, sum over K∈𝒯, and use discrete integration by parts (14):

$$\begin{array}{ll} 0 & =\sum_{K\in 𝒯}\frac{m\left(K\right)}{D}\frac{w_{0,K}^{2}}{\Delta t}+\sum_{\sigma =K|L\in {ℰ}_{\mathrm{int}}}\tau_{\sigma }{\left(w_{0,K}-w_{0,L}\right)}^{2}\\ & \;+\sum_{\sigma =K|L\in {ℰ}_{\mathrm{int}}}\tau_{\sigma }\left(\left(u_{0,K}-u_{0,L}\right)|u_{0,K}-u_{0,L}|-\left(v_{0,K}-v_{0,L}\right)|v_{0,K}-v_{0,L}|\;\right)\left(w_{0,K}-w_{0,L}\right). \end{array}$$

The first two terms on the right‐hand side are clearly nonnegative. We infer from the elementary inequality (*y*|*y*| − *z*|*z*|)(*y* − *z*) ≥ 0 for *y*, *z* ∈ ℝ, which is a consequence of the monotonicity of *z* ↦ *z*|*z*|, that the third term is nonnegative, too. Consequently, the three terms must vanish and this implies that *w*_0, *K*_ = 0 for all K∈𝒯. This shows the uniqueness for *u*_0_.

*Step 2: uniqueness for u*_*i*_. Let *u*_0_ be the uniquely determined solution from the previous step and let $u^{k}=\left(u_{1}^{k},\ldots ,u_{n}^{k}\right)$ and $v^{k}=\left(v_{1}^{k},\ldots ,v_{n}^{k}\right)$ be two solutions to (15). Similarly as in Gajewski 7, we introduce the semimetric

$$\begin{array}{l} d_{\epsilon }\left(u^{k},v^{k}\right)=\sum_{K\in 𝒯}m\left(K\right)\sum_{i=1}^{n}H_{1}^{\epsilon }\left(u_{i,K}^{k},v_{i,K}^{k}\right),\;\text{where}\\ H_{1}^{\epsilon }\left(a,b\right)=h_{\epsilon }\left(a\right)+h_{\epsilon }\left(b\right)-2h_{\epsilon }\left(\frac{a+b}{2}\right) \end{array}$$

and *h*_*ϵ*_(*z*) = (*z* + *ϵ*)(log(*z* + *ϵ*) − 1) + 1. The parameter *ϵ* > 0 is needed since $u_{i,K}^{k}$ or $v_{i,K}^{k}$ may vanish and then the logarithm of $u_{i,K}^{k}$ or $v_{i,K}^{k}$ may be undefined. The objective is to verify that $\lim_{\epsilon \to 0}d_{\epsilon }\left(u^{k},v^{k}\right)=0$ by estimating the discrete time derivative of the semimetric, implying that *u* ^*k*^ = *v* ^*k*^.

First, we write

$$d_{\epsilon }\left(u^{k},v^{k}\right)-d_{\epsilon }\left(u^{k-1},v^{k-1}\right)=\sum_{K\in 𝒯}m\left(K\right)\sum_{i=1}^{n}\left(H_{1}^{\epsilon }\left(u_{i,K}^{k},v_{i,K}^{k}\right)-H_{1}^{\epsilon }(u_{i,K}^{k-1},v_{i,K}^{k-1})\right).$$

The function $H_{1}^{\epsilon }$ is convex since

$$D^{2}H_{1}^{\epsilon }\left(a,b\right)=\frac{1}{\left(a+\epsilon \right)\left(b+\epsilon \right)\left(a+b+2\epsilon \right)}\left(\begin{array}{cc} {\left(b+\epsilon \right)}^{2} & -\left(a+\epsilon \right)\left(b+\epsilon \right)\\ -\left(a+\epsilon \right)\left(b+\epsilon \right) & {\left(a+\epsilon \right)}^{2} \end{array}\right).$$

Therefore, a Taylor expansion of $H_{1}^{\epsilon }$ around $\left(u_{i,K}^{k},v_{i,K}^{k}\right)$ leads to

$$\begin{array}{ll} \frac{1}{△t} & \left(d_{\epsilon }\left(u^{k},v^{k}\right)-d_{\epsilon }(u^{k-1},v^{k-1})\right)\\ & \leq \sum_{K\in 𝒯}\frac{m\left(K\right)}{△t}\sum_{i=1}^{n}\left\{{\mathrm{DH}}_{1}^{\epsilon }\left(u_{i,K}^{k},v_{i,K}^{k}\right)\left(\left(\begin{array}{c} u_{i,K}^{k}\\ v_{i,K}^{k} \end{array}\right)-\left(\begin{array}{c} u_{i,K}^{k-1}\\ v_{i,K}^{k-1} \end{array}\right)\right)\right\}\\ & =\sum_{i=1}^{n}\sum_{K\in 𝒯}m\left(K\right)\frac{u_{i,K}^{k}-u_{i,K}^{k-1}}{△t}\left(h_{\epsilon^{\prime }}\left(u_{i,K}^{k}\right)-h_{\epsilon }^{\prime }\left(\frac{u_{i,K}^{k}+v_{i,K}^{k}}{2}\right)\right)\\ & \;+\sum_{i=1}^{n}\sum_{K\in 𝒯}m\left(K\right)\frac{v_{i,K}^{k}-v_{i,K}^{k-1}}{△t}\left(h_{\epsilon }^{\prime }\left(v_{i,K}^{k}\right)-h_{\epsilon }^{\prime }\left(\frac{u_{i,K}^{k}+v_{i,K}^{k}}{2}\right)\right). \end{array}$$

We insert the scheme (21) and (22) and use discrete integration by parts:

$$\frac{1}{△t}\left(d_{\epsilon }\left(u^{k},v^{k}\right)-d_{\epsilon }(u^{k-1},v^{k-1})\right)\leq S_{1}^{k}+S_{2}^{k}+\epsilon S_{3}^{k},$$

where

$$\begin{array}{cc} & S_{1}^{k}=-D\sum_{i=1}^{n}\sum_{\sigma =K|L\in \epsilon_{\mathrm{int}}}\tau_{\sigma }u_{0,\sigma }^{k}\{\left(u_{i,K}^{k}-u_{i,L}^{k}\right)\left(\log\left(u_{i,K}^{k}+\epsilon \right)-\log\left(u_{i,L}^{k}+\epsilon \right)\right)\\ & +\left(v_{i,K}^{k}-v_{i,L}^{k}\right)\left(\log\left(v_{i,K}^{k}+\epsilon \right)-\log\left(v_{i,L}^{k}+\epsilon \right)\right)\\ & -2\left(\frac{u_{i,K}^{k}+v_{i,K}^{k}}{2}-\frac{u_{i,L}^{k}+v_{i,L}^{k}}{2}\right)\left(\log\left(\frac{u_{i,K}^{k}+v_{i,K}^{k}}{2}+\epsilon \right)-\log\left(\frac{u_{i,L}^{k}+v_{i,L}^{k}}{2}+\epsilon \right)\right)\},\\ & S_{2}^{k}=D\sum_{i=1}^{n}\sum_{\sigma =K|L\in \epsilon_{\mathrm{int}}}\tau_{\sigma }\left(u_{0,K}^{k}-u_{0,L}^{k}\right)\{\left(u_{i,\sigma }^{k}+\epsilon \right)\left(\log\left(u_{i,K}^{k}+\epsilon \right)-\log\left(u_{i,L}^{k}+\epsilon \right)\right)\\ & +\left(v_{i,\sigma }^{k}+\epsilon \right)\left(\log\left(v_{i,K}^{k}+\epsilon \right)-\log\left(v_{i,L}^{k}+\epsilon \right)\right)\\ & -2\left(\frac{u_{i,\sigma }^{k}+v_{i,\sigma }^{k}}{2}+\epsilon \right)\left(\log\left(\frac{u_{i,K}^{k}+v_{i,K}^{k}}{2}+\epsilon \right)-\log\left(\frac{u_{i,L}^{k}+v_{i,L}^{k}}{2}+\epsilon \right)\right)\}, \end{array}$$

$$\begin{array}{l} S_{3}^{k}=-D\sum_{i=1}^{n}\sum_{\sigma =K|L\in \epsilon_{\mathrm{int}}}\tau_{\sigma }\left(u_{0,K}^{k}-u_{0,L}^{k}\right)\{\left(\log\left(u_{i,K}^{k}+\epsilon \right)-\log\left(u_{i,L}^{k}+\epsilon \right)\right)\\ +\left(\log\left(v_{i,K}^{k}+\epsilon \right)-\log\left(v_{i,L}^{k}+\epsilon \right)\right)\\ -2\left(\log\left(\frac{u_{i,K}^{k}+v_{i,K}^{k}}{2}+\epsilon \right)-\log\left(\frac{u_{i,L}^{k}+v_{i,L}^{k}}{2}+\epsilon \right)\right)\}. \end{array}$$

We claim that $S_{1}^{k}\leq 0$ and $S_{2}^{k}\leq 0$. Indeed, with the definition $H_{2}^{\epsilon }\left(a,b\right)=\left(a-b\right)\left(\log\left(a+\epsilon \right)-\log\left(b+\epsilon \right)\right)$, we can reformulate $S_{1}^{k}$ as

$$\begin{array}{ll} S_{1}^{k} & =-D\sum_{i=1}^{n}\sum_{\sigma =K|L\in {ℰ}_{\mathrm{int}}}\tau_{\sigma }u_{0,\sigma }^{k}\left\{H_{2}^{\epsilon }\left(u_{i,K}^{k},u_{i,L}^{k}\right)+H_{2}^{\epsilon }\left(v_{i,K}^{k},v_{i,L}^{k}\right)\right.\\ & \left.\;-2H_{2}^{\epsilon }\left(\frac{u_{i,K}^{k}+v_{i,K}^{k}}{2},\frac{u_{i,L}^{k}+v_{i,L}^{k}}{2}\right)\right\}. \end{array}$$

The Hessian of $H_{2}^{\epsilon }$,

$$D^{2}H_{2}^{\epsilon }\left(a,b\right)=\left(\begin{array}{cc} \frac{a+b+2\epsilon }{{\left(a+\epsilon \right)}^{2}} & -\frac{a+b+2\epsilon }{\left(a+\epsilon \right)\left(b+\epsilon \right)}\\ -\frac{a+b+2\epsilon }{\left(a+\epsilon \right)\left(b+\epsilon \right)} & \frac{a+b+2\epsilon }{{\left(b+\epsilon \right)}^{2}} \end{array}\right),$$

is positive semidefinite. Therefore, performing a Taylor expansion up to second order, we see that $S_{1}^{k}\leq 0$.

Next, we show that $S_{2}^{k}\leq 0$. For this, we assume without loss of generality for some fixed *σ* = *K*|*L* that $u_{0,K}^{k}\leq u_{0,L}^{k}$. By definition of the scheme, $u_{i,\sigma }^{k}=u_{i,K}^{k}$ and $v_{i,\sigma }^{k}=v_{i,K}^{k}$. Set $H_{3}^{\epsilon }\left(a,b\right)=\left(a+\epsilon \right)\left(\log\left(a+\epsilon \right)-\log\left(b+\epsilon \right)\right)$. The term in the curly bracket in $S_{2}^{k}$ then takes the form

(31) $$\left(u_{0,K}^{k}-u_{0,L}^{k}\right)\left\{H_{3}^{\epsilon }\left(u_{i,K}^{k},u_{i,L}^{k}\right)+H_{3}^{\epsilon }\left(v_{i,K}^{k},v_{i,L}^{k}\right)-2H_{3}^{\epsilon }\left(\frac{u_{i,K}^{k}+v_{i,K}^{k}}{2},\frac{u_{i,L}^{k}+v_{i,L}^{k}}{2}\right)\right\}.$$

The Hessian of $H_{3}^{\epsilon }$,

$$D^{2}H_{3}^{\epsilon }\left(a,b\right)=\left(\begin{array}{cc} \frac{1}{a+\epsilon } & -\frac{1}{b+\epsilon }\\ -\frac{1}{b+\epsilon } & \frac{a+\epsilon }{{\left(b+\epsilon \right)}^{2}} \end{array}\right),$$

is also positive semidefinite, showing that (31) is nonpositive as $u_{0,K}^{k}-u_{0,L}^{k}\leq 0$. If $u_{0,K}^{k}>u_{0,L}^{k}$, both factors of the product (31) change their sign, so that we arrive at the same conclusion. Hence, $S_{2}^{k}\leq 0$. We conclude that

$$d_{\epsilon }\left(u^{k},v^{k}\right)-d_{\epsilon }\left(u^{k-1},v^{k-1}\right)\leq \epsilon \Delta {\mathrm{tS}}_{3}^{k}.$$

Since *d*_*ϵ*_(*u* ^0^, *v* ^0^) = 0, we find after resolving the recursion that

$$d_{\epsilon }\left(u^{k},v^{k}\right)\leq \epsilon △t\sum_{\ell =1}^{k}S_{3}^{\ell }.$$

As the densities *u* ${}_{i,K}^{\ell }$ are nonnegative and bounded by 1 for all K∈𝒯, for all *ℓ* ≥ 0 and for all 1 ≤ *i* ≤ *n*, it is clear that $\sum_{\ell =1}^{k}\epsilon S_{3}^{\ell }\to 0$ when *ϵ* → 0. Then, we may perform the limit *ϵ* → 0 in the previous inequality yielding *d*_*ϵ*_(*u* ^*k*^, *v* ^*k*^) → 0. A Taylor expansion as in Zamponi and Jüngel [8, end of Section 6] shows that $d_{\epsilon }\left(u^{k},v^{k}\right)\geq \frac{1}{8}\sum_{K\in 𝒯}m\left(K\right)\sum_{i=1}^{n}{\left(u_{i,K}^{k}-v_{i,K}^{k}\right)}^{2}$. We infer that *u* ^*k*^ = *v* ^*k*^, finishing the proof.

## 4 DISCRETE ENTROPY INEQUALITY AND UNIFORM ESTIMATES

### 4.1 Discrete entropy inequality

First, we prove (25).

Proof of Theorem 2 The idea is to multiply (15) by $\log\left(u_{i,K}^{k,\epsilon }/u_{0,K}^{k,\epsilon }\right)$, where $u_{i,K}^{k,\epsilon }≔u_{i,K}^{k}+\epsilon$ for *i* = 0, …, *n*. The regularization is necessary to avoid issues when the concentrations vanish. After this multiplication, we sum the equations over *i* = 1, …, *n* and K∈𝒯 and use discrete integration by parts to obtain

(32) $$\begin{array}{ll} 0 & =\sum_{K\in 𝒯}\frac{m\left(K\right)}{△\mathrm{tD}}\sum_{i=1}^{n}\left(u_{i,K}^{k}-u_{i,K}^{k-1}\right)\log\frac{u_{i,K}^{k,\epsilon }}{u_{0,K}^{k,\epsilon }}\\ & +\sum_{\sigma =K|L\in {ℰ}_{\mathrm{int}}}\tau_{\sigma }\left(u_{0,\sigma }^{k}\left(u_{i,K}^{k}-u_{i,L}^{k}\right)-u_{i,\sigma }^{k}\left(u_{0,K}^{k}-u_{0,L}^{k}\right)\right)\left(\log\frac{u_{i,K}^{k,\epsilon }}{u_{0,K}^{k,\epsilon }}-\log\frac{u_{i,L}^{k,\epsilon }}{u_{0,L}^{k,\epsilon }}\right)\\ & =A_{0}+\sum_{\sigma =K|L\in {ℰ}_{\mathrm{int}}}\tau_{\sigma }\left(A_{1}+A_{2}+B_{1}+B_{2}\right), \end{array}$$

where

$$\begin{array}{ll} A_{0} & =\sum_{K\in 𝒯}\frac{m\left(K\right)}{△\mathrm{tD}}\sum_{i=0}^{n}\left(u_{i,K}^{k,\epsilon }-u_{i,K}^{k-1,\epsilon }\right)\log u_{i,K}^{k,\epsilon },\\ A_{1} & =\sum_{i=1}^{n}u_{0,\sigma }^{k}\left(u_{i,K}^{k,\epsilon }-u_{i,L}^{k,\epsilon }\right)\left(\log u_{i,K}^{k,\epsilon }-\log u_{i,L}^{k,\epsilon }\right),\\ A_{2} & =-\sum_{i=1}^{n}u_{0,\sigma }^{k}\left(u_{i,K}^{k}-u_{i,L}^{k}\right)\left(\log u_{0,K}^{k,\epsilon }-\log u_{0,L}^{k,\epsilon }\right),\\ B_{1} & =-\sum_{i=1}^{n}u_{i,\sigma }^{k}\left(u_{0,K}^{k}-u_{0,L}^{k}\right)\left(\log u_{i,K}^{k,\epsilon }-\log u_{i,L}^{k,\epsilon }\right),\\ B_{2} & =\sum_{i=1}^{n}u_{i,\sigma }^{k}\left(u_{0,K}^{k}-u_{0,L}^{k}\right)\left(\log u_{0,K}^{k,\epsilon }-\log u_{0,L}^{k,\epsilon }\right). \end{array}$$

The convexity of *h*(*z*) = *z*(log*z* − 1) + 1 implies the inequality *h*(*u*) − *h*(*v*) ≤ *h* ^*'*^(*u*)(*u* − *v*) for all *u*, *v* ∈ ℝ. Consequently,

$$A_{0}\geq \sum_{K\in 𝒯}\frac{m\left(K\right)}{△\mathrm{tD}}\sum_{i=0}^{n}\left(u_{i,K}^{k,\epsilon }\left(\log u_{i,K}^{k,\epsilon }-1\right)-u_{i,K}^{k-1,\epsilon }\left(\log u_{i,K}^{k-1,\epsilon }-1\right)\right).$$

In order to estimate the remaining terms, we recall two elementary inequalities. Let *y*, *z* > 0. Then, by the Cauchy–Schwarz inequality,

(33) $${\left(\sqrt{y}-\sqrt{z}\right)}^{2}={\left(\int_{z}^{y}\frac{\mathrm{ds}}{2\sqrt{s}}\right)}^{2}\leq \int_{z}^{y}\frac{\mathrm{ds}}{4}\int_{z}^{y}\frac{\mathrm{ds}}{s}=\frac{1}{4}\left(y-z\right)\left(\log y-\log z\right),$$

and by the concavity of the logarithm,

(34) $$y\left(\log y-\log z\right)\geq y-z\geq z\left(\log y-\log z\right).$$

Inequality (33) shows that

$$A_{1}\geq 4\sum_{i=1}^{n}u_{0,\sigma }^{k}\left({\left(u_{i,K}^{k,\epsilon }\right)}^{1/2}-{\left(u_{i,L}^{k,\epsilon }\right)}^{1/2}\right).$$

We use the definition of $u_{0,K}^{k}=1-\sum_{i=1}^{n}u_{i,K}^{k}$ in *A*_2_ to find that

$$A_{2}=u_{0,\sigma }^{k}\left(u_{0,K}^{k}-u_{0,L}^{k}\right)\left(\log u_{0,K}^{k,\epsilon }-\log u_{0,L}^{k,\epsilon }\right).$$

We rewrite *B*_1_ by using the abbreviation $u_{i,\sigma }^{k,\epsilon }=u_{i,\sigma }^{k}+\epsilon$:

$$\begin{array}{ll} B_{1} & =-\sum_{i=1}^{n}u_{i,\sigma }^{k,\epsilon }\left(u_{0,K}^{k}-u_{0,L}^{k}\right)\left(\log u_{i,K}^{k,\epsilon }-\log u_{i,L}^{k,\epsilon }\right)\\ & \;+\epsilon \sum_{i=1}^{n}\left(u_{0,K}^{k}-u_{0,L}^{k}\right)\left(\log u_{i,K}^{k,\epsilon }-\log u_{i,L}^{k,\epsilon }\right)\\ & ≕B_{11}+\epsilon B_{12}. \end{array}$$

We apply inequality (34) to *B*_11_. Indeed, if $u_{0,K}^{k}\leq u_{0,L}^{k}$, we have $u_{i,\sigma }^{k}=u_{i,K}^{k}$ and we use the first inequality in (34). If $u_{0,K}^{k}>u_{0,L}^{k}$ then $u_{i,\sigma }^{k}=u_{i,L}^{k}$ and we employ the second inequality in (34). In both cases, it follows that

$$\begin{array}{ll} B_{11} & \geq -\sum_{i=1}^{n}\left(u_{0,K}^{k}-u_{0,L}^{k}\right)\left(u_{i,K}^{k,\epsilon }-u_{i,L}^{k,\epsilon }\right)\\ & =-\left(u_{0,K}^{k}-u_{0,L}^{k}\right)\sum_{i=1}^{n}\left(u_{i,K}^{k,\epsilon }-u_{i,L}^{k,\epsilon }\right)={\left(u_{0,K}^{k}-u_{0,L}^{k}\right)}^{2}. \end{array}$$

Finally, we consider *B*_2_. In view of Assumption (A3), Equation (23) gives

(35) $$\sum_{i=1}^{n}u_{i,\sigma }^{k}=1-\min\left\{u_{0,K}^{k},u_{0,L}^{k}\right\}\geq 1-u_{0,\sigma }^{k},$$

and therefore, by (33),

$$\begin{array}{ll} B_{2} & \geq \left(1-u_{0,\sigma }^{k}\right)\left(u_{0,K}^{k,\epsilon }-u_{0,L}^{k,\epsilon }\right)\left(\log u_{0,K}^{k,\epsilon }-\log u_{0,L}^{k,\epsilon }\right)\\ & \geq 4{\left({\left(u_{0,K}^{k,\epsilon }\right)}^{1/2}-{\left(u_{0,L}^{k,\epsilon }\right)}^{1/2}\right)}^{2}-u_{0,\sigma }^{k}\left(u_{0,K}^{k}-u_{0,L}^{k}\right)\left(\log u_{0,K}^{k,\epsilon }-\log u_{0,L}^{k,\epsilon }\right). \end{array}$$

The last expression cancels with *A*_2_ such that

$$A_{2}+B_{2}\geq 4{\left({\left(u_{0,K}^{k,\epsilon }\right)}^{1/2}-{\left(u_{0,L}^{k,\epsilon }\right)}^{1/2}\right)}^{2}.$$

Putting together the estimates for *A*_0_, *A*_1_, *B*_1_, and *A*_2_ + *B*_2_, we deduce from (32) that

$$\begin{array}{ll} & \sum_{K\in 𝒯}\frac{m\left(K\right)}{△t}\sum_{i=0}^{n}u_{i,K}^{k,\epsilon }\left(\log u_{i,K}^{k,\epsilon }-1\right)-\sum_{K\in 𝒯}\frac{m\left(K\right)}{△t}\sum_{i=1}^{n}u_{i,K}^{k-1,\epsilon }\left(\log u_{i,K}^{k-1,\epsilon }-1\right)\\ & \;+D\sum_{\sigma =K|L\in {ℰ}_{\mathrm{int}}}\tau_{\sigma }\left\{4\sum_{i=1}^{n}u_{0,\sigma }{\left({\left(u_{i,K}^{k,\epsilon }\right)}^{1/2}-{\left(u_{i,L}^{k,\epsilon }\right)}^{1/2}\right)}^{2}\right.\\ & \left.\;+4{\left({\left(u_{0,K}^{k,\epsilon }\right)}^{1/2}-{\left(u_{0,L}^{k,\epsilon }\right)}^{1/2}\right)}^{2}+{\left(u_{0,K}^{k}-u_{0,L}^{k}\right)}^{2}\right\}\\ & \;\leq -\mathrm{\epsilon D}\left(u_{0,K}^{k}-u_{0,L}^{k}\right)\sum_{i=1}^{n}\left(\log u_{i,K}^{k,\epsilon }-\log u_{i,L}^{k,\epsilon }\right). \end{array}$$

Since the right‐hand side converges to zero as *ϵ* → 0, we infer that (25) holds.

### 4.2 A priori estimates

For the proof of the convergence result, we need estimates uniform in the mesh size $h\left(𝒯\right)$ and time step △*t*. The scheme provides uniform *L*^∞^ bounds. Further bounds are derived from the discrete entropy inequality of theorem 2. We introduce the discrete time derivative for functions $v\in {ℋ}_{𝒯,△t}$ by

(36) $$\partial_{t}^{△t}v^{k}=\frac{v^{k}-v^{k-1}}{△t},\;k=1,\ldots ,N.$$

Lemma 7 (A priori estimates). *Let (H1)–(H4) and (A1)–(A3) hold. The solution u to scheme* (21), (22) *satisfies the following uniform estimates*:

(37) $${\left\|u_{0}^{1/2}\right\|}_{1,𝒯,△t}+{\left\|u_{0}^{1/2}u_{i}\right\|}_{1,𝒯,△t}\leq C,\;i=1,\ldots ,n,$$

(38) $$\sum_{k=1}^{N}△t{\left\|\partial_{t}^{△t}u_{i}^{k}\right\|}_{-1,𝒯}^{2}\leq C,\;i=0,\ldots ,n,$$

*where the constant C > 0 is independent of the mesh* 𝒯 *and time step size* △*t*.

We claim that estimates (37) follow from the discrete entropy inequality (25). Indeed, we sum (25) over *k* = 1, …, *N* to obtain

$$\begin{array}{ll} H^{N} & +D\sum_{k=1}^{N}△t\sum_{\sigma =K|L\in {ℰ}_{\mathrm{int}}}\tau_{\sigma }\left(4\sum_{i=1}^{n}u_{0,\sigma }^{k}{\left({\left(u_{i,K}^{k}\right)}^{1/2}-{\left(u_{i,L}^{k}\right)}^{1/2}\right)}^{2}\right.\\ & \left.+4{\left({\left(u_{0,K}^{k}\right)}^{1/2}-{\left(u_{0,L}^{k}\right)}^{1/2}\right)}^{2}+{\left(u_{0,K}^{k}-u_{0,L}^{k}\right)}^{2}\right)\leq H^{0}. \end{array}$$

Since the entropy at time *t* = 0 is bounded independently of the discretization, we infer immediately the bound for $u_{0}^{1/2}$ in ${ℋ}_{𝒯,△t}$. For the bound on $u_{0}^{1/2}u_{i}$ in ${ℋ}_{𝒯,△t}$, we observe that

$$\begin{array}{l} {\left(u_{0,K}^{k}\right)}^{1/2}u_{i,K}^{k}-{\left(u_{0,L}^{k}\right)}^{1/2}u_{i,L}^{k}=u_{i,K}\left({\left(u_{0,K}^{k}\right)}^{1/2}-{\left(u_{0,L}^{k}\right)}^{1/2}\right)\\ +{\left(u_{0,L}^{k}\right)}^{1/2}\left({\left(u_{i,K}^{k}\right)}^{1/2}+{\left(u_{i,L}^{k}\right)}^{1/2}\right)\left({\left(u_{i,K}^{k}\right)}^{1/2}-{\left(u_{i,L}^{k}\right)}^{1/2}\right). \end{array}$$

Therefore, together with the *L*^∞^ bounds on *u*_*i*_,

$$\begin{array}{l} \sum_{\sigma =K|L\in {ℰ}_{\mathrm{int}}}\tau_{\sigma }{\left({\left(u_{0,K}^{k}\right)}^{1/2}u_{i,K}^{k}-{\left(u_{0,L}^{k}\right)}^{1/2}u_{i,L}^{k}\right)}^{2}\\ \leq \sum_{\sigma =K|L\in {ℰ}_{\mathrm{int}}}\tau_{\sigma }{\left({\left(u_{0,K}^{k}\right)}^{1/2}-{\left(u_{0,L}^{k}\right)}^{1/2}\right)}^{2}+2\sum_{\sigma =K|L\in {ℰ}_{\mathrm{int}}}\tau_{\sigma }u_{0,\sigma }^{k}{\left({\left(u_{i,K}^{k}\right)}^{1/2}-{\left(u_{i,L}^{k}\right)}^{1/2}\right)}^{2}. \end{array}$$

Then, summing over *k* = 0, …, *N* and using the estimates from the entropy inequality, we achieve the bound on $u_{0}^{1/2}u_{i}$.

It remains to prove estimate (38). To this end, let $\phi \in {ℋ}_{𝒯}$ be such that ${\left\|\phi \right\|}_{1,𝒯}=1$ and let *k* ∈ {1, …, *N*} and *i* ∈ {1, …, *n*}. We multiply the Scheme (21) by Φ_*K*_ and we sum over K∈𝒯. Using successively discrete integration by parts, the rewriting of the numerical fluxes (24), the Cauchy–Schwarz inequality, and the *L*^∞^ bounds on *u*_*i*_, we compute

$$\begin{array}{ll} & \sum_{K\in 𝒯}\frac{m\left(K\right)}{△t}\left(u_{i,K}^{k}-u_{i,K}^{k-1}\right)\phi_{K}\\ & \;=D\sum_{\sigma =K|L\in {ℰ}_{\mathrm{int}}}\tau_{\sigma }{\left(u_{0,\sigma }^{k}\right)}^{1/2}\left({\left(u_{0,K}^{k}\right)}^{1/2}u_{i,K}^{k}-{\left(u_{0,L}^{k}\right)}^{1/2}u_{i,L}^{k}\right)\left(\phi_{K}-\phi_{L}\right) \end{array}$$

$$\begin{array}{ll} & \;-D\sum_{\sigma =K|L\in {ℰ}_{\mathrm{int}}}\tau_{\sigma }\left({\left(u_{0,K}^{k}\right)}^{1/2}-{\left(u_{0,L}^{k}\right)}^{1/2}\right)\\ & \;\times u_{i,\sigma }^{k}\left({\left(u_{0,\sigma }^{k}\right)}^{1/2}+2\frac{{\left(u_{0,K}^{k}\right)}^{1/2}+{\left(u_{0,L}^{k}\right)}^{1/2}}{2}\right)\left(\phi_{K}-\phi_{L}\right)\\ & \;\leq D{\left(\sum_{\sigma =K|L\in {ℰ}_{\mathrm{int}}}\tau_{\sigma }{\left({\left(u_{0,K}^{k}\right)}^{1/2}u_{i,K}^{k}-{\left(u_{0,L}^{k}\right)}^{1/2}u_{i,L}^{k}\right)}^{2}\right)}^{1/2}{\left(\sum_{\sigma =K|L\in {ℰ}_{\mathrm{int}}}\tau_{\sigma }{\left(\phi_{K}-\phi_{L}\right)}^{2}\right)}^{1/2}\\ & \;+3D{\left(\sum_{\sigma =K|L\in {ℰ}_{\mathrm{int}}}\tau_{\sigma }{\left({\left(u_{0,K}^{k}\right)}^{1/2}-{\left(u_{0,L}^{k}\right)}^{1/2}\right)}^{2}\right)}^{1/2}{\left(\sum_{\sigma =K|L\in {ℰ}_{\mathrm{int}}}\tau_{\sigma }{\left(\phi_{K}-\phi_{L}\right)}^{2}\right)}^{1/2}. \end{array}$$

This shows that, for *i* = 1, …, *n*,

$$\sum_{k=1}^{N}△t{\left\|\frac{u_{i}^{k}-u_{i}^{k-1}}{△t}\right\|}_{-1,𝒯}^{2}\leq 2D^{2}\sum_{k=1}^{N}△t\left({\left\|{\left(u_{0}^{k}\right)}^{1/2}u_{i}^{k}\right\|}_{1,𝒯}^{2}+9{\left\|{\left(u_{0}^{k}\right)}^{1/2}\right\|}_{1,𝒯}^{2}\right)\leq C,$$

as a consequence of (37). The estimate for $\Delta t^{-1}\left(u_{0}^{k}-u_{0}^{k-1}\right)=-\Delta t^{-1}\sum_{i=1}^{n}\left(u_{i}^{k}-u_{i}^{k-1}\right)$ follows from those for *i* = 1, …, *n*, completing the proof.

## 5 CONVERGENCE OF THE SCHEME

In this section, we establish the convergence of the sequence of approximate solutions, constructed in theorem 1, to a weak solution to (1), that is, we prove theorem 3.

### 5.1 Compactness of the approximate solutions

In order to achieve the convergence in the fluxes, we proceed as in Chainais‐Hillairet and coworkers 22 by defining the approximate gradient on a dual mesh. For *σ* = *K* ∣ *L* ∈ ℰ_int_, we define the new cell *T*_*KL*_ as the cell with the vertexes *x*_*K*_, *x*_*L*_ and those of *σ*. For *σ* ∈ ℰ_ext_ ∩ ℰ_*K*_, we define *T*_*Kσ*_ as the cell with vertex *x*_*K*_ and those of *σ*. Then Ω can be decomposed as

$$\bar{\Omega }=\underset{K\in 𝒯}{⋃}\{(\underset{L\in {𝒩}_{K}}{⋃}{\bar{T}}_{\mathrm{KL}})⋃(\underset{\sigma \in {ℰ}_{\mathrm{ext},K}}{⋃}{\bar{T}}_{K\sigma })\},$$

where ${𝒩}_{K}$ denotes the set of neighboring cells of *K*. The discrete gradient $\nabla_{𝒯,△t}v$ on Ω_*T*_: = Ω × (0, *T*) for piecewise constant functions $v\in {ℋ}_{𝒯,△t}$ is defined by

(39) $$\nabla_{𝒯,△t}v\left(x,t\right)=\left\{\begin{array}{ll} \frac{m\left(\sigma \right)\left(v_{L}^{k}-v_{K}^{k}\right)}{m\left(T_{\mathrm{KL}}\right)}n_{\mathrm{KL}}\; & \text{for}\;x\in T_{\mathrm{KL}},\;t\in \left(t^{k},t^{k+1}\right),\\ 0\; & \text{for}\;x\in T_{K\sigma },\;t\in \left(t^{k},t^{k+1}\right), \end{array}\right.$$

where **n**_*KL*_ denotes the unit normal on *σ* = *K*|*L* oriented from *K* to *L*. To simplify the notation, we set $\nabla_{m}≔\nabla_{{𝒯}_{m},△t_{m}}$. The solution to the approximate scheme (21) and (22) is called *u*_0, *m*_, *u*_1, *m*_, …, *u*_*n*, *m*_.

Lemma 8 *There exist functions* u_0_ ∈ L^∞^(Ω_T_) ∩ L^2^(0, T; H^*1*^(Ω)) *and* *u*_1_, …, *u*_*n*_ ∈ *L*^∞^(Ω_*T*_) *such that, possibly for subsequences, as* m → ∞,

(40) $$u_{0,m}\to u_{0},\;u_{0,m}^{1/2}\to u_{0}^{1/2}\;\text{strongly in}\;L^{2}\left(\Omega_{T}\right),$$

(41) $$\nabla_{m}u_{0,m}⇀\nabla u_{0},\;\nabla_{m}u_{0,m}^{1/2}⇀\nabla u_{0}^{1/2}\;\text{weakly in}\;L^{2}\left(\Omega_{T}\right),$$

(42) $$u_{0,m}^{1/2}u_{i,m}\to u_{0}^{1/2}u_{i}\;\text{strongly in}\;L^{2}\left(\Omega_{T}\right),$$

(43) $$\nabla_{m}\left(u_{0,m}^{1/2}u_{i,m}\right)⇀\nabla \left(u_{0}^{1/2}u_{i}\right)\;\text{weakly in}\;L^{2}\left(\Omega_{T}\right),$$

where i ∈ {1, …, n}.

First, we claim that (*u*_0, *m*_) is uniformly bounded in ${ℋ}_{𝒯,△t}$. Indeed, by the *L*^∞^ bounds and estimate (37),

(44) $$\begin{array}{ll} {\left\|u_{0,m}\right\|}_{1,𝒯,\Delta t}^{2} & =\sum_{k=1}^{N}\Delta t\left(\sum_{\sigma =K|L\in {ℰ}_{\mathrm{int}}}\tau_{\sigma }{\left(u_{0,K}^{k}-u_{0,L}^{k}\right)}^{2}+\sum_{K\in 𝒯}m\left(K\right){\left(u_{0,K}^{k}\right)}^{2}\right)\\ & =\sum_{k=1}^{N}\Delta t\left(\sum_{\sigma =K|L\in {ℰ}_{\mathrm{int}}}\tau_{\sigma }{\left({\left(u_{0,K}^{k}\right)}^{1/2}+{\left(u_{0,L}^{k}\right)}^{1/2}\right)}^{2}{\left({\left(u_{0,K}^{k}\right)}^{1/2}-{\left(u_{0,L}^{k}\right)}^{1/2}\right)}^{2}\right.\\ & \left.+\sum_{K\in 𝒯}m\left(K\right){\left(u_{0,K}^{k}\right)}^{2}\right)\\ & \leq 4{\left\|u_{0,m}\right\|}_{L^{\infty }\left(\Omega_{T}\right)}{\left\|u_{0,m}^{1/2}\right\|}_{1,𝒯,\Delta t}^{2}+{\left\|u_{0,m}\right\|}_{L^{2}\left(\Omega_{T}\right)}^{2}\leq C. \end{array}$$

By estimate (38), $\left(\partial_{t}^{△t}u_{0,m}\right)$ is uniformly bounded. Therefore, by the discrete Aubin–Lions lemma (see lemma 9 in the appendix), we conclude the existence of a subsequence (not relabeled) such that the first convergence in (40) holds. The strong convergence implies (up to a subsequence) that *u*_0, *m*_ → *u*_0_ pointwise in Ω_*T*_ and consequently $u_{0,m}^{1/2}\to u_{0}^{1/2}$ pointwise in Ω_*T*_. Thus, together with the *L*^∞^ bound for $u_{0,m}^{1/2}$, we infer the second convergence in (40).

The convergences in (41) are a consequence of the uniform estimates (37) and (44) and the compactness result in Eymard and coworkers [16, proof of Theor. 10.3]. Applying the discrete Aubin–Lions lemma of “degenerate” type (see lemma 10 in the appendix) to $y_{m}=u_{0,m}^{1/2}$ and *z*_*m*_ = *u*_*i*, *m*_ for fixed *i* ∈ {1, …, *n*}, we deduce convergence (42). Finally, convergence (43) is a consequence of (42) and the weak compactness of $\left(u_{0,m}^{1/2}u_{i,m}\right)$, thanks to the uniform bound in (37).

### 5.2 The limit *m* → ∞

We finish the proof of theorem 3 by verifying that the limit function *u* = (*u*_1_, …, *u*_*n*_), as defined in lemma 8, is a weak solution in the sense of the theorem.

Let $\phi \in C_{0}^{\infty }(\bar{\Omega }\times [0,T))$ and let *m* ∈ ℕ be large enough such that $\mathrm{supp}\phi \subset \bar{\Omega }\times \left[0,\left(N_{m}-1\right)△t_{m}\right)$ (recall that *T* = *N*_*m*_△*t*_*m*_). For the limit, we follow the strategy used, for instance, in Chainais‐Hillairet and coworkers 22 and introduce the following notations:

$$\begin{array}{ll} F_{10}\left(m\right) & =-\int_{0}^{T}\int_{\Omega }u_{i,m}\partial_{t}\text{ϕdxdt}-\int_{\Omega }u_{i,m}\left(0\right)\phi \left(0\right)\mathrm{dx},\\ F_{20}\left(m\right) & =\int_{0}^{T}\int_{\Omega }u_{0,m}^{1/2}\nabla_{m}\left(u_{0,m}^{1/2}u_{i,m}\right)\nabla \text{ϕdxdt},\\ F_{30}\left(m\right) & =3\int_{0}^{T}\int_{\Omega }u_{0,m}^{1/2}u_{i,m}\nabla_{m}\left(u_{0,m}^{1/2}\right)\nabla \text{ϕdxdt}. \end{array}$$

The convergence results of lemma 8 show that, as *m* → ∞,

(45) $$\begin{array}{ll} F_{10}\left(m\right)+{\mathrm{DF}}_{20}\left(m\right)-{\mathrm{DF}}_{30}\left(m\right) & \to -\int_{0}^{T}\int_{\Omega }u_{i}\partial_{t}\text{ϕdxdt}-\int_{\Omega }u_{i}^{0}\phi \left(0\right)\mathrm{dx}\\ & +D\int_{0}^{T}\int_{\Omega }\left(u_{0}^{1/2}\nabla \left(u_{0}^{1/2}u_{i}\right)-3u_{0}^{1/2}u_{i}\nabla u_{0}^{1/2}\right)\text{dxdt}. \end{array}$$

Next, setting $\phi_{K}^{k}=\phi \left(x_{K},t^{k}\right)$, we multiply Scheme (21) by $△t_{m}\phi_{K}^{k-1}$ and sum over $K\in {𝒯}_{m}$ and *k* = 1, …, *N*_*m*_. Then

(46) $$F_{1}\left(m\right)+{\mathrm{DF}}_{2}\left(m\right)-{\mathrm{DF}}_{3}\left(m\right)=0,$$

where, omitting the subscript *m* from now on to simplify the notation,

$$\begin{array}{ll} F_{1}\left(m\right) & =\sum_{k=1}^{N}\sum_{K\in 𝒯}m\left(K\right)\left(u_{i,K}^{k}-u_{i,K}^{k-1}\right)\phi_{K}^{k-1},\\ F_{2}\left(m\right) & =\sum_{k=1}^{N}\Delta t\sum_{K\in 𝒯}\sum_{\sigma \in {ℰ}_{K,\mathrm{int}}}\tau_{\sigma }{\left(u_{0,\sigma }^{k}\right)}^{1/2}\left({\left(u_{0,K}^{k}\right)}^{1/2}u_{i,K}^{k}-{\left(u_{0,L}^{k}\right)}^{1/2}u_{i,L}^{k}\right)\phi_{K}^{k-1},\\ F_{3}\left(m\right) & =\sum_{k=1}^{N}\Delta t\sum_{K\in 𝒯}\sum_{\sigma \in {ℰ}_{K,\mathrm{int}}}\tau_{\sigma }\left({\left(u_{0,K}^{k}\right)}^{1/2}-{\left(u_{0,L}^{k}\right)}^{1/2}\right)\\ & \times u_{i,\sigma }^{k}\left({\left(u_{0,\sigma }^{k}\right)}^{1/2}+2\frac{{\left(u_{0,K}^{k}\right)}^{1/2}+{\left(u_{0,L}^{k}\right)}^{1/2}}{2}\right)\phi_{K}^{k-1}. \end{array}$$

The aim is to show that *F*_*i*0_(*m*) − *F*_*i*_(*m*) → 0 as *m* → ∞ for *i* = 1, 2, 3. Then, because of (46), *F*_10_(*m*) + *DF*_20_(*m*) − *DF*_30_(*m*) → 0, which finishes the proof. We start by verifying that *F*_10_(*m*) − *F*_1_(*m*) → 0. For this, we rewrite *F*_1_(*m*) and *F*_10_(*m*), using $\phi_{K}^{N}=0$:

$$\begin{array}{ll} F_{1}\left(m\right) & =\sum_{k=1}^{N}\sum_{K\in 𝒯}m\left(K\right)u_{i,K}^{k}\left(\phi_{K}^{k-1}-\phi_{K}^{k}\right)-\sum_{K\in 𝒯}m\left(K\right)\phi_{K}^{0}u_{i,K}^{0},\\ & =-\sum_{k=1}^{N}\sum_{K\in 𝒯}\int_{t^{k-1}}^{t^{k}}\int_{K}u_{i,K}^{k}\partial_{t}\phi \left(x_{K},t\right)\text{dxdt}-\sum_{K\in 𝒯}\int_{K}u_{i,K}^{0}\phi \left(x_{K},0\right)\mathrm{dx},\\ F_{10}\left(m\right) & =-\sum_{k=1}^{N}\sum_{K\in 𝒯}\int_{t^{k-1}}^{t^{k}}\int_{K}u_{i,K}^{k}\partial_{t}\phi \left(x,t\right)\text{dxdt}-\sum_{K\in 𝒯}\int_{K}u_{i,K}^{0}\phi \left(x,0\right)\mathrm{dx}. \end{array}$$

In view of the regularity of *ϕ* and the uniform *L*^∞^ bound on *u*_*i*_, we find that

$$|F_{10}\left(m\right)-F_{1}\left(m\right)|\leq \mathrm{CT}m\left(\Omega \right){\left\|\phi \right\|}_{C^{2}}h\left({𝒯}_{m}\right)\to 0\;\mathrm{as}\;m\to \infty .$$

Using discrete integration by parts, the second integral becomes

$$\begin{array}{ll} F_{2}\left(m\right) & =\sum_{k=1}^{N}\Delta t\sum_{\sigma =K|L\in {ℰ}_{\mathrm{int}}}\tau_{\sigma }{\left(u_{0,\sigma }^{k}\right)}^{1/2}\left({\left(u_{0,K}^{k}\right)}^{1/2}u_{i,K}^{k}-{\left(u_{0,L}^{k}\right)}^{1/2}u_{i,L}^{k}\right)\left(\phi_{K}^{k-1}-\phi_{L}^{k-1}\right)\\ & =F_{21}\left(m\right)+F_{22}\left(m\right), \end{array}$$

where we have decomposed ${\left(u_{0,\sigma }^{k}\right)}^{1/2}={\left(u_{0,K}^{k}\right)}^{1/2}+\left({\left(u_{0,\sigma }^{k}\right)}^{1/2}-{\left(u_{0,K}^{k}\right)}^{1/2}\right)$, i.e.

$$\begin{array}{ll} F_{21}\left(m\right) & =\sum_{k=1}^{N}\Delta t\sum_{\sigma =K|L\in {ℰ}_{\mathrm{int}}}\tau_{\sigma }{\left(u_{0,K}^{k}\right)}^{1/2}\left({\left(u_{0,K}^{k}\right)}^{1/2}u_{i,K}^{k}-{\left(u_{0,L}^{k}\right)}^{1/2}u_{i,L}^{k}\right)\left(\phi_{K}^{k-1}-\phi_{L}^{k-1}\right), \end{array}$$

$$\begin{array}{ll} F_{22}\left(m\right) & =\sum_{k=1}^{N}\Delta t\sum_{\sigma =K|L\in {ℰ}_{\mathrm{int}}}\tau_{\sigma }\left({\left(u_{0,\sigma }^{k}\right)}^{1/2}-{\left(u_{0,K}^{k}\right)}^{1/2}\right)\left({\left(u_{0,K}^{k}\right)}^{1/2}u_{i,K}^{k}-{\left(u_{0,L}^{k}\right)}^{1/2}u_{i,L}^{k}\right)\times \left(\phi_{K}^{k-1}-\phi_{L}^{k-1}\right). \end{array}$$

Furthermore, we write *F*_20_(*m*) = *G*_1_(*m*) + *G*_2_(*m*), where

$$\begin{array}{ll} G_{1}\left(m\right) & =\sum_{k=1}^{N}\sum_{\sigma =K|L\in {ℰ}_{\mathrm{int}}}\frac{m\left(\sigma \right)}{m\left(T_{\mathrm{KL}}\right)}{\left(u_{0,K}^{k}\right)}^{1/2}\left({\left(u_{0,K}^{k}\right)}^{1/2}u_{i,K}^{k}-{\left(u_{0,L}^{k}\right)}^{1/2}u_{i,L}^{k}\right)\\ & \times \int_{t^{k-1}}^{t^{k}}\int_{T_{\mathrm{KL}}}\nabla \phi \left(x,t\right)\cdot n_{K\sigma }\text{dxdt},\\ G_{2}\left(m\right) & =\sum_{k=1}^{N}\sum_{\sigma =K|L\in {ℰ}_{\mathrm{int}}}\frac{m\left(\sigma \right)}{m\left(T_{\mathrm{KL}}\right)}\left({\left(u_{0,L}^{k}\right)}^{1/2}-{\left(u_{0,K}^{k}\right)}^{1/2}\right)\left({\left(u_{0,K}^{k}\right)}^{1/2}u_{i,K}^{k}-{\left(u_{0,L}^{k}\right)}^{1/2}u_{i,L}^{k}\right)\\ & \times \int_{t^{k-1}}^{t^{k}}\int_{T_{\mathrm{KL}}\cap L}\nabla \phi \left(x,t\right)\cdot n_{K\sigma }\text{dxdt}. \end{array}$$

The aim is to show that *F*_21_(*m*) − *G*_1_(*m*) → 0, *F*_22_(*m*) → 0, and *G*_2_(*m*) → 0. This implies that

$$\begin{array}{ll} |F_{20}\left(m\right)-F_{2}\left(m\right)| & =\left|\left(G_{1}\left(m\right)+G_{2}\left(m\right)\right)-\left(F_{21}\left(m\right)+F_{22}\left(m\right)\right)\right|\\ & \leq |G_{1}-F_{21}|+|G_{2}|+|F_{22}|\to 0. \end{array}$$

First we notice that, due to the admissibility of the mesh and the regularity of *ϕ*, by taking the mean value over *T*_*KL*_,

(47) $$\left|\int_{t^{k-1}}^{t^{k}}\left(\frac{\phi_{K}^{k-1}-\phi_{L}^{k-1}}{d_{\sigma }}-\frac{1}{m\left(T_{\mathrm{KL}}\right)}\int_{T_{\mathrm{KL}}}\nabla \phi \left(x,t\right)\cdot n_{K\sigma }\right)\mathrm{dt}|\leq C△\mathrm{th}\left(𝒯\right),\;\right.$$

where the constant *C* > 0 only depends on *ϕ*. It yields

$$\begin{array}{ll} |F_{21}\left(m\right)-G_{1}\left(m\right)| & \leq \mathrm{Ch}\left(𝒯\right)\sum_{k=1}^{N}\Delta t\sum_{\sigma =K|L\in {ℰ}_{\mathrm{int}}}m\left(\sigma \right)\left|{\left(u_{0,K}^{k}\right)}^{1/2}u_{i,K}-{\left(u_{0,L}^{k}\right)}^{1/2}u_{i,L}\right|\\ & \leq \mathrm{Ch}\left(𝒯\right){\left\|u_{0}^{1/2}u_{i}\right\|}_{1,𝒯,\Delta t}{\left(Tm\left(\Omega \right)\right)}^{1/2}, \end{array}$$

where the last estimate follows from the Cauchy–Schwarz inequality. This proves that |*F*_21_(*m*) − *G*_1_(*m*)| → 0 as *m* → ∞.

It remains to analyze the expressions *F*_22_(*m*) and *G*_2_(*m*). To this end, we remark that $d_{\sigma }\leq h\left(𝒯\right)$ and hence, together with the regularity of *ϕ*, and the Cauchy‐Schwarz inequality,

$$\begin{array}{ll} |F_{22}\left(m\right)| & \leq \sum_{k=1}^{N}△t\sum_{\sigma =K|L\in {ℰ}_{\mathrm{int}}}\tau_{\sigma }\left|{\left(u_{0,\sigma }^{k}\right)}^{1/2}-{\left(u_{0,K}^{k}\right)}^{1/2}\right|\left|{\left(u_{0,K}^{k}\right)}^{1/2}u_{i,K}^{k}-{\left(u_{0,L}^{k}\right)}^{1/2}u_{i,L}^{k}\right|\\ & \times \frac{|\phi_{K}^{k-1}-\phi_{L}^{k-1}|}{d_{\sigma }}d_{\sigma }\\ & \leq \mathrm{Ch}\left(𝒯\right){\left\|\phi \right\|}_{C^{1}}\sum_{k=1}^{N}△t\sum_{\sigma =K|L\in {ℰ}_{\mathrm{int}}}\tau_{\sigma }\left|{\left(u_{0,\sigma }^{k}\right)}^{1/2}-{\left(u_{0,K}^{k}\right)}^{1/2}\right|\\ & \times \left|{\left(u_{0,K}^{k}\right)}^{1/2}u_{i,K}^{k}-{\left(u_{0,L}^{k}\right)}^{1/2}u_{i,L}^{k}\right|\\ & \leq \mathrm{Ch}\left(𝒯\right){\left\|\phi \right\|}_{C^{1}}{\left\|u_{0}^{1/2}\right\|}_{1,𝒯,△t}{\left\|u_{0}^{1/2}u_{i}\right\|}_{1,𝒯,△t}\leq \mathrm{Ch}\left(𝒯\right), \end{array}$$

The term *G*_2_(*m*) can be estimated in a similar way.

Finally, we need to show that |*F*_30_(*m*) − *F*_3_(*m*)| → 0. The is completely analogous to the previous arguments, since

$$\begin{array}{ll} |3{\left(u_{0,K}^{k}\right)}^{1/2} & u_{i,K}^{k}-u_{i,\sigma }^{k}\left({\left(u_{0,\sigma }^{k}\right)}^{1/2}+2\frac{{\left(u_{0,K}^{k}\right)}^{1/2}+{\left(u_{0,L}^{k}\right)}^{1/2}}{2}\right)|\\ & \leq C\left({\left(u_{0,\sigma }^{k}\right)}^{1/2}|u_{i,K}^{k}-u_{i,L}^{k}|+|{\left(u_{0,K}^{k}\right)}^{1/2}-{\left(u_{0,L}^{k}\right)}^{1/2}|\;\right). \end{array}$$

Summarizing, we have proved that |*F*_*i*0_(*m*) − *F*_*i*_(*m*)| → 0 for *i* = 1, 2, 3, and since *F*_1_(*m*) + *DF*_2_(*m*) − *DF*_3_(*m*) = 0, the convergence (45) shows that *u* solves (27). This completes the proof of Theorem 3.

## 6 NUMERICAL EXPERIMENTS

**Figure 1:**
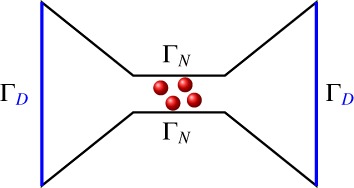
Schematic picture of the ion channel Ω used for the simulations. Dirichlet boundary conditions are prescribed on Γ_*D*_ (blue), homogeneous Neumann boundary conditions on Γ_*N*_ (black). The red circles represent the confined *O* ^1/2−^ ions [Color figure can be viewed at wileyonlinelibrary.com]

We present numerical simulations of a calcium‐selective ion channel in two space dimensions to illustrate the dynamical behavior of the ion transport model. Numerical simulations in one space dimension can be found in Burger and coworkers 23 for stationary solutions and in Gerstenmayer and Jüngel 6 for transient solutions. The channel is modeled as in Gillespie coworkers 24. The selectivity of the channel is obtained by placing some confined oxygen ions (O^1/2−^) inside the channel region. These ions contribute to the permanent charge density *f* = −*u*_*ox*_/2 in the Poisson equation, but also to the total sum of the concentrations. We consider three further types of ions: calcium (Ca^2+^, *u*_1_), sodium (Na^+^, *u*_2_), and chloride (Cl^−^, *u*_3_). While the concentrations of these ion species satisfy the evolution equations (1), the oxygen concentration is constant in time and given by the piecewise linear function

$$u_{\mathrm{ox}}\left(x,y\right)=u_{\mathrm{ox},\max}\times \left\{\begin{array}{ll} 1\; & \text{for}\;0.45\leq x\leq 0.55,\\ 10\left(x-0.35\right)\; & \text{for}\;0.35\leq x\leq 0.45,\\ 10\left(0.65-x\right)\; & \text{for}\;0.55\leq x\leq 0.65,\\ 0\; & \text{else}, \end{array}\right.$$

where the scaled maximal oxygen concentration equals *u*_ox, max_ = (*N*_*A*_/*u*_typ_) · 52mol/L, where *N*_*A*_ ≈ 6.022 × 10^23^ mol^−1^ is the Avogadro constant and *u*_*typ*_ = 3.7037 × 10^25^ *L* ^−1^ the typical concentration (taken from [23, tab. 1]). This gives *u*_ox, max_ ≈ 0.84. The solvent concentration is computed according to $u_{0}=1-\sum_{i=1}^{3}u_{i}-u_{\mathrm{ox}}$. The physical parameters used in our simulations are taken from [23, tab. 1], and the channel geometry is depicted in Figure 1. The boundary conditions are chosen as in Burger and coworkers [23, Section 5].

The simulations are performed with the full set of Equations (1), (2) without assuming (A1)–(A3). The finite‐volume Scheme (15), (16), (17), (18), (19), (20) is implemented using MATLAB, version R2015a. The nonlinear system defined by the implicit scheme is solved with a full Newton method in the variables *u*_0_, *u*_1_, *u*_2_, *u*_3_, Φ for every time step. The computations are done with a fixed time step size △*t* = 10^−3^ until the stationary state is approximately reached, that is, until the discrete *L* ^2^ norm between the solutions at two consecutive time steps is smaller than 10^−12^. We employ an admissible mesh with 4,736 elements generated by the MATLAB command initmesh, which produces Delauney meshes. As initial data, piecewise linear functions that connect the boundary values are chosen for the ion concentrations, while the initial potential is computed from the Poisson equation using the initial concentrations as charge density.

**Figure 2:**
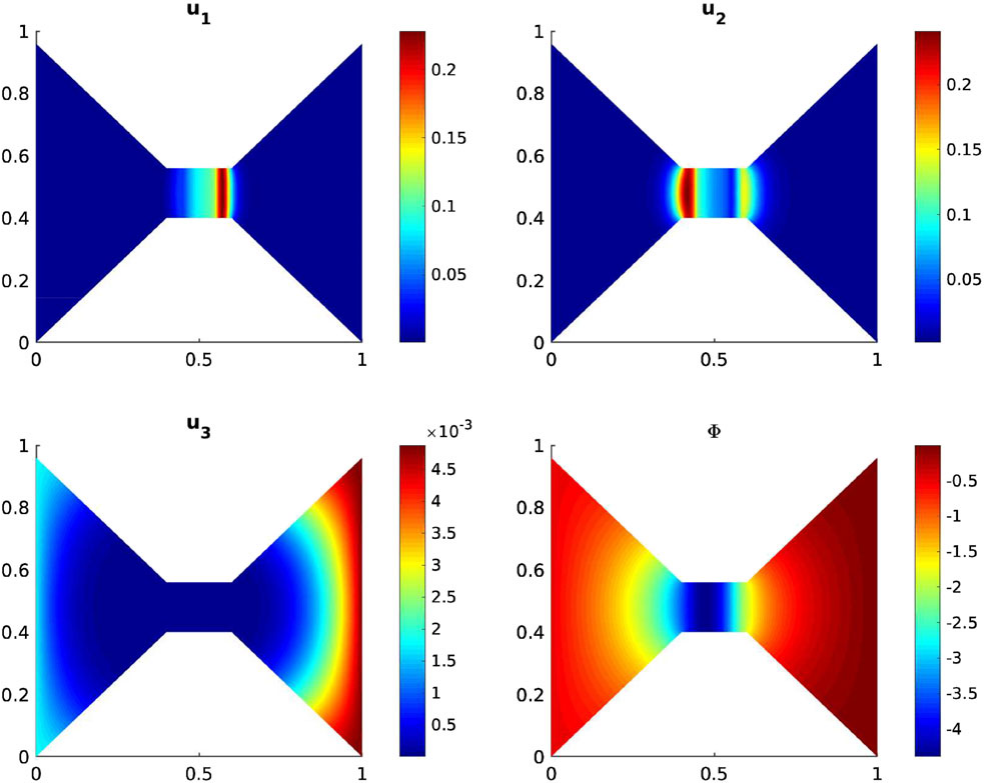
Scaled concentrations of calcium, sodium, and chloride ions and electric potential after 50 time steps [Color figure can be viewed at wileyonlinelibrary.com]

**Figure 3:**
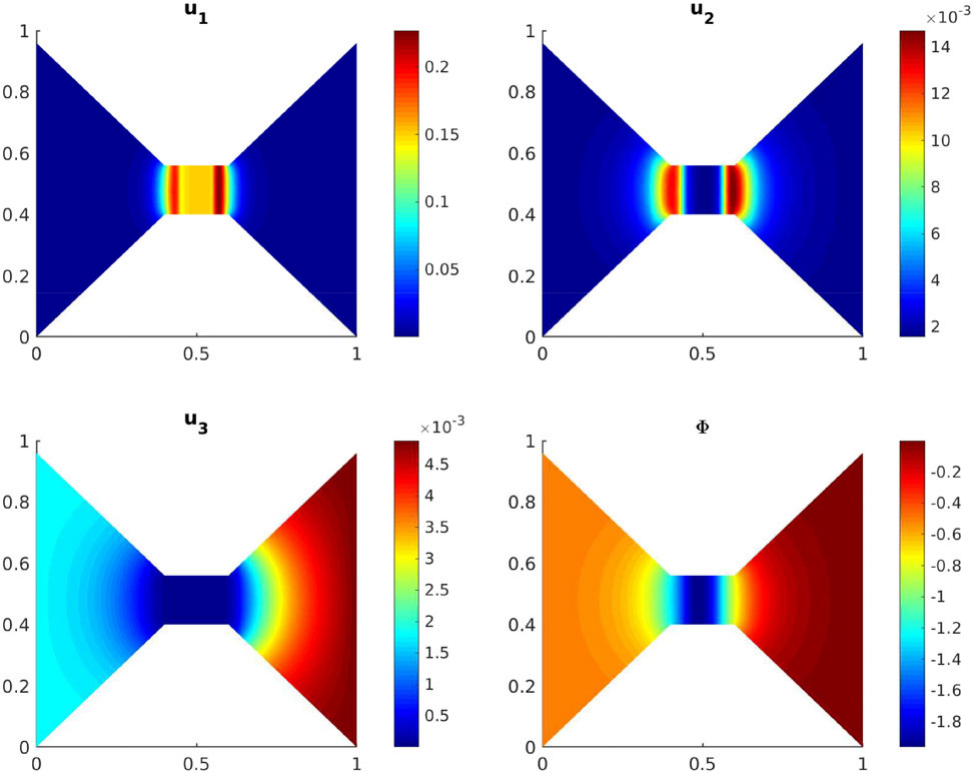
Scaled concentrations of calcium, sodium, and chloride ions and electric potential after 1,400 time steps (close to equilibrium) [Color figure can be viewed at wileyonlinelibrary.com]

Figures 2 and 3 show the concentration profiles and the electric potential after 50 and 1,400 time steps, respectively. The equilibrium is approximately reached after 1,653 time steps. The profiles depicted in Figure 3 are already very close to the stationary state and correspond qualitatively well to the one‐dimensional stationary profiles presented in Burger and coworkers 23. We observe that during the evolution, sodium inside the channel is replaced by the stronger positively charged calcium ions. For higher initial calcium concentrations, the calcium selectivity of the channel acts immediately.

**Figure 4:**
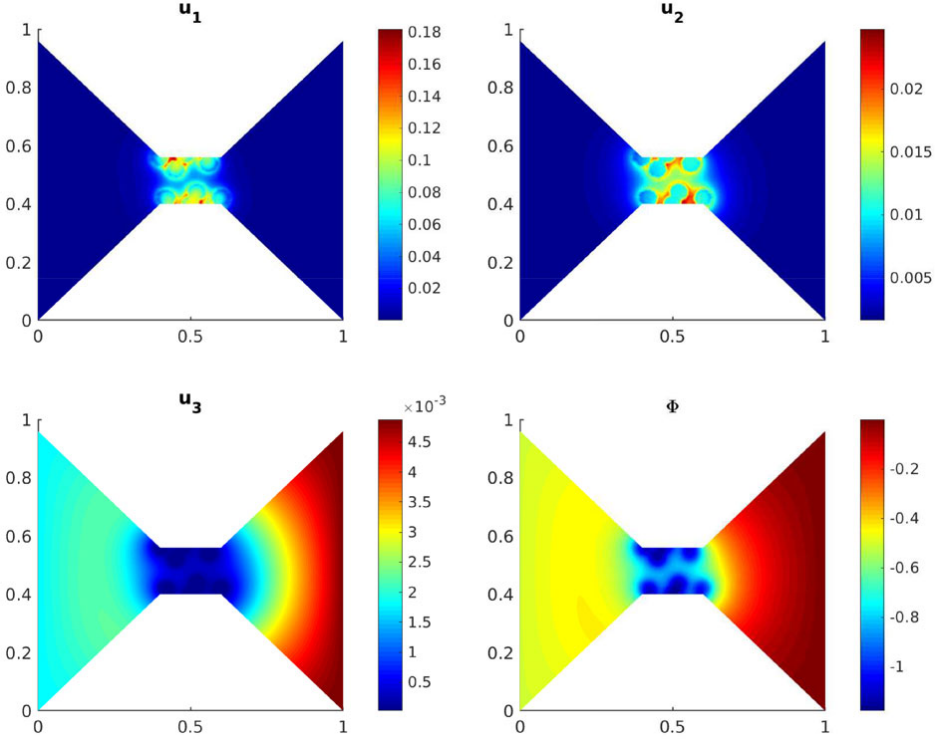
Scaled concentrations of calcium, sodium, and chloride ions and electric potential in the asymmetric equilibrium state [Color figure can be viewed at wileyonlinelibrary.com]

The piecewise linear oxygen concentration used in our simulations is clearly not optimal for describing the complex structure of a selectivity filter. As an attempt for a more realistic model, we represent the eight confined oxygen ions as fixed circular discs with a scaled radius of 0.03 (corresponding to 15 nm) and centers inside the channel region. On these discs, the oxygen concentration is set to *u*_ox, max_, otherwise it equals zero. Figure 4 shows the concentration profiles and the potential in the equilibrium state. Again, we observe that the calcium ions are selected over the sodium ions, while chloride is removed from the channel.

**Figure 5:**
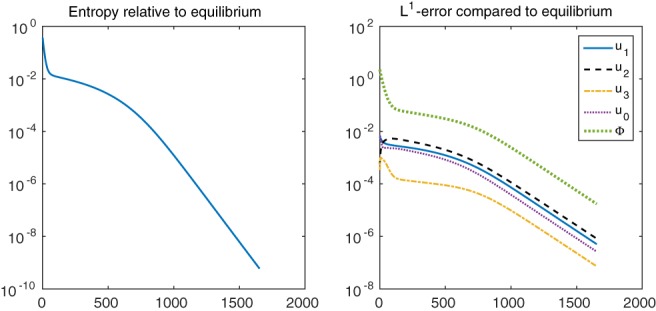
Relative entropy (left) and discrete *L* ^1^ error relative to the equilibrium (right) over the number of time steps using *λ* ^2^ = 4.68 × 10^−4^ [Color figure can be viewed at wileyonlinelibrary.com]

The simulations suggest that the solution tends towards a steady state as *t* → ∞. The large‐time behavior can be quantified by computing the relative entropy *E* ^*k*^ with respect to the stationary solution, where

$$E^{k}=\sum_{K\in 𝒯}m\left(K\right)\sum_{i=0}^{n}u_{i,K}^{k}\log\left(\frac{u_{i,K}^{k}}{u_{i,K}^{\infty }}\right)+\frac{\lambda^{2}}{2}\sum_{\sigma \in ℰ}\tau_{\sigma }D_{K,\sigma }{\left(\Phi^{k}-\Phi^{\infty }\right)}^{2}$$

and $\left(u_{i,K}^{\infty },\Phi^{\infty }\right)$ is the steady state determined from the boundary data. Figure 5 shows that the relative entropy as well as the discrete *L* ^1^ norms of the concentrations and electric potential decay with exponential rate. Interestingly, after some initial phase, the convergence is rather slow and increases after this intermediate phase. This phase can be explained by the degeneracy at *u*_0_ = 0, which causes a small entropy production slowing down diffusion. Indeed, as shown in Gerstenmayer and Jüngel 6 for the one‐dimensional setting, a small change in the oxygen concentration may prolong the intermediate phase of slow convergence drastically.

**Figure 6:**
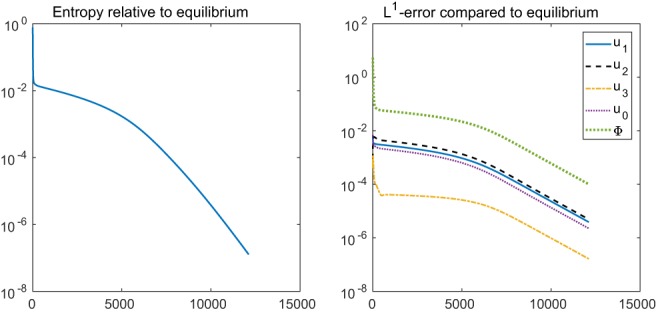
Relative entropy (left) and discrete *L* ^1^ error relative to the equilibrium (right) over the number of time steps using *λ* ^2^ = 2 × 10^−4^ [Color figure can be viewed at wileyonlinelibrary.com]

The scaled permittivity constant *λ* in the Poisson equation has the value *λ* ^2^ = 4.68 × 10^−4^. Therefore, the drift term is moderately convective (in the sense that the modulus of the electric field |∇Φ| is moderately large). Figure 6 shows the entropy decay and the *L* ^1^ error for a smaller value of *λ* = 2 × 10^−4^. It turns out that the scheme is still entropy dissipative, but the entropy decay is much slower and the *L* ^1^ error decreases with smaller rate.

**Figure 7:**
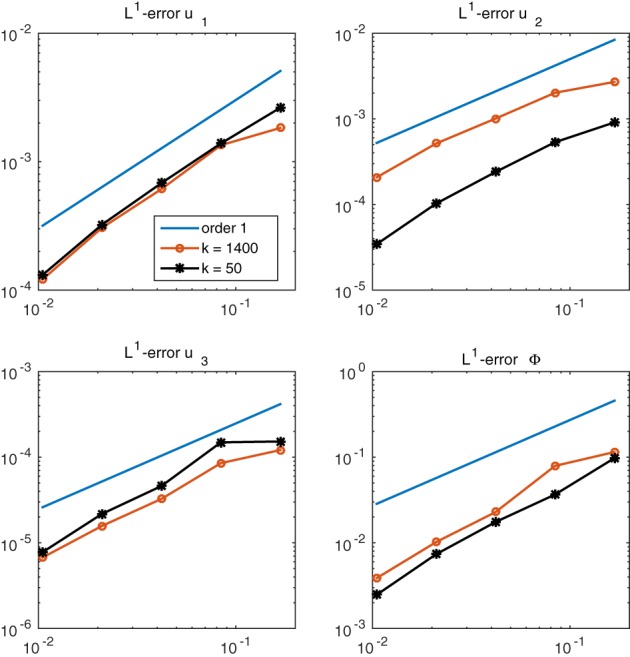
Discrete *L* ^1^ error relative to the reference solution at two different time steps over the mesh size $h\left(𝒯\right)$ [Color figure can be viewed at wileyonlinelibrary.com]

Since Assumptions (A1)–(A3) are not satisfied in our test case, the convergence result of theorem 3 cannot be applied here. However, we still observe convergence of the numerical solutions. As the exact solution is not known explicitly, we compute a reference solution on a very fine mesh with 75,776 elements and mesh size $h\left(𝒯\right)\approx 0.01$. This mesh is obtained from the coarse mesh by a regular refinement, dividing the triangles into four triangles of the same shape. The reference solution is compared to approximate solutions on coarser nested meshes. In Figure 7, the errors in the discrete *L* ^1^ norm between the reference solution and the solutions on the coarser meshes at two fixed time steps *k* = 50 and *k* = 1,400 are plotted. We clearly observe the expected first‐order convergence in space.

## APPENDIX 1 AUXILIARY RESULTS

We prove two versions of discrete Aubin–Lions lemmas. The first one is a consequence of [18, Theor. 3.4], the second one extends Lemma 13 in 5 to the discrete case. The latter result is new. Recall that Ω_*T*_ = Ω × (0, *T*), $\nabla_{m}=\nabla_{{𝒯}_{m},△t_{m}}$ is the discrete gradient defined in (39), and $\partial_{t}^{△t}$ is the discrete time derivative defined in (36). Lemma 9 (Discrete Aubin–Lions). *Let* ${\left\|\cdot \right\|}_{1,{𝒯}_{m}}$ *be the norm on* ${ℋ}_{{𝒯}_{m}}$ *defined in* (11) *with the dual norm* ${\left\|\cdot \right\|}_{-1,{𝒯}_{m}}$ *given by* (12), *and let* $\left(u_{m}\right)\subset {ℋ}_{{𝒯}_{m},△t_{m}}$ *be a sequence of piecewise constants in time functions with values in* ${ℋ}_{{𝒯}_{m}}$ *satisfying*

$$\sum_{k=1}^{N_{m}}△t\left({\left\|u_{m}^{k}\right\|}_{1,{𝒯}_{m}}^{2}+{\left\|\partial_{t}^{△t_{m}}u_{m}^{k}\right\|}_{-1,{𝒯}_{m}}^{2}\right)\leq C,$$

*where C > 0 is independent of the size of the mesh and the time step size. Then there exists a subsequence, which is not relabeled, such that, as* m → ∞,

$$\begin{array}{ll} u_{m}\to u & \;\text{strongly in}\;L^{2}\left(\Omega_{T}\right),\\ \nabla_{m}u_{m}⇀\nabla u & \;\text{weakly in}\;L^{2}\left(\Omega_{T}\right). \end{array}$$

The result is a consequence of Theorem 3.4 in 18. To apply this theorem, we have to show that the discrete norms ${\left\|\cdot \right\|}_{1,{𝒯}_{m}}$ and ${\left\|\cdot \right\|}_{-1,{𝒯}_{m}}$ satisfy the assumptions of Lemma 3.1 in 18:

1. For any sequence $\left(v_{m}\right)\subset {ℋ}_{{𝒯}_{m}}$ such that there exists *C* > 0 with ${\left\|v_{m}\right\|}_{1,{𝒯}_{m}}\leq C$ for all *m* ∈ ℕ, there exists *v* ∈ *L* ^2^(Ω) such that, up to a subsequence, *v*_*m*_ → *v* in *L* ^2^(Ω).
2. If *v*_*m*_ → *v* strongly in *L* ^2^(Ω) and ${\left\|v_{m}\right\|}_{-1,{𝒯}_{m}}\to 0$ as *m* → ∞, then *v* = 0.

The first property is proved in, for instance, [21, Lem. 5.6]. Here, we need assumption (10) on the mesh. The second property can be replaced, according to [18, Rem. 6], by the condition that ${\left\|\cdot \right\|}_{1,{𝒯}_{m}}$ and ${\left\|\cdot \right\|}_{-1,{𝒯}_{m}}$ are dual norms with respect to the *L* ^2^(Ω) norm, which is the case here. We infer that there exists a subsequence of (*u*_*m*_), which is not relabeled, such that *u*_*m*_ → *u* strongly in *L* ^2^(Ω_*T*_). The weak convergence of the discrete gradients can be proved as in Lemma 4.4 in Chainais‐Hillairet and coworkers 22. Indeed, the boundedness of (∇_*m*_ *u*_*m*_) in *L* ^2^ implies the convergence to some function *χ* ∈ *L* ^2^(Ω_*T*_) (up to a subsequence). In order to show that *χ* = ∇*u*, it remains to verify that for all test functions $\phi \in C_{0}^{\infty }\left(\Omega_{T};{\mathbb{R}}^{d}\right)$,

$$\int_{0}^{T}\int_{\Omega }\nabla_{m}u_{m}\cdot \text{ϕdxdt}+\int_{0}^{T}\int_{\Omega }u_{m}\;\mathrm{div}\;\text{ϕdxdt}\to 0\;\mathrm{as}\;m\to \infty .$$

This limit follows from the definition of ∇_*m*_ *u*_*m*_ and the regularity of the mesh. We refer to [22, Lem. 4.4] for details.
Lemma 10 (Discrete Aubin–Lions of “degenerate” type). *Let (y*_m_ *) and (z*_m_ *) be sequences in* ${ℋ}_{{𝒯}_{m},△t_{m}}$ *which are bounded in* *L*^∞^(Ω_T_) *and let (y*_m_ *) be relatively compact in L* ^2^(Ω_T_ *), i.e., up to a subsequence, y*_m_ → *y strongly in L* ^2^ *(*Ω_T_ *) and* z_m_⇀^*^z *weakly* in* L^∞^*(*Ω_T_*)*. *Furthermore, suppose that, for some constant C > 0 independent of m,*

$$\sum_{k=1}^{N_{m}}△t_{m}\left({\left\|y_{m}^{k}\right\|}_{1,{𝒯}_{m}}^{2}+{\left\|y_{m}^{k}z_{m}^{k}\right\|}_{1,{𝒯}_{m}}^{2}+{\left\|\partial_{t}^{△t_{m}}z_{m}^{k}\right\|}_{-1,{𝒯}_{m}}^{2}\right)\leq C.$$

*Then there exists a subsequence which is not relabeled such that y*_m_z_m_ → *yz strongly in L* ^2^ *(*Ω_T_ *) as* m → ∞.
The idea of the proof is to use the Kolmogorov‐Riesz theorem [25, Theor. 4.26] as in the continuous case; see [2, Section 4.4] or [5, Lem. 13]. The discrete case, however, makes necessary some changes in the calculations. We need to show that

(48) $$\lim_{\left(\xi ,\tau \right)\to 0}\int_{0}^{T-\tau }\int_{\omega }{\left(\left(y_{m}z_{m}\right)(x+\xi ,t+\tau )-\left(y_{m}z_{m}\right)(x,t)\right)}^{2}\text{dxdt}=0$$

uniformly in *m*, where *ω* ⊂ Ω satisfies *x* + *ξ* ∈ Ω for all *x* ∈ *ω*. First, we separate the space and time translation:

$$\begin{array}{l} \int_{0^{T}}-\tau \int_{\omega }{\left(\left(y_{m}z_{m}\right)(x+\xi ,t+\tau )-\left(y_{m}z_{m}\right)(x,t)\right)}^{2}\text{dxdt}\\ \leq 2\int_{0}^{T-\tau }\int_{\omega }{\left(\left(y_{m}z_{m}\right)(x+\xi ,t+\tau )-\left(y_{m}z_{m}\right)(x,t+\tau )\right)}^{2}\text{dxdt}\\ +2\int_{0}^{T-\tau }\int_{\omega }{\left(\left(y_{m}z_{m}\right)(x,t+\tau )-\left(y_{m}z_{m}\right)(x,t)\right)}^{2}\text{dxdt}≕I_{1}+I_{2}. \end{array}$$

For the estimate of *I*_1_, we apply a result for space translations of piecewise constant functions *v* with uniform bounds in the discrete *H* ^1^(Ω) norm, namely

$${\left\|v\left(\cdot +\xi \right)-v\right\|}_{L^{2}\left(\Omega \right)}^{2}\leq |\xi |\left(\;|\xi |+\mathrm{Ch}\left(𝒯\right)\right){\left\|v\right\|}_{1,𝒯}^{2}$$

for appropriate *ξ*, where *C* > 0 only depends on Ω [26, Lem. 4]. This shows that

$$I_{1}\leq C_{1}|\xi |\left(\;|\xi |+\mathrm{Ch}\left({𝒯}_{m}\right)\right)$$

converges to zero as *ξ* → 0 uniformly in *m*. For the second integral *I*_2_, we write

$$\begin{array}{ll} I_{2} & \leq 4\int_{0}^{T-\tau }\int_{\omega }z_{m}{\left(x,t+\tau \right)}^{2}{\left(y_{m}\left(x,t+\tau \right)-y_{m}(x,t)\right)}^{2}\text{dxdt}\\ & \;+4\int_{0}^{T-\tau }\int_{\omega }y_{m}{\left(x,t\right)}^{2}{\left(z_{m}\left(x,t+\tau \right)-z_{m}(x,t)\right)}^{2}\text{dxdt}≕I_{21}+I_{22}. \end{array}$$

The *L*^∞^ bounds on *z*_*m*_ give

$$I_{21}\leq C\int_{0}^{T-\tau }\int_{\omega }{\left(y_{m}\left(x,t+\tau \right)-y_{m}(x,t)\right)}^{2}\text{dxdt}.$$

By assumption, the sequence (*y*_*m*_) is relatively compact in *L* ^2^(Ω_*T*_). Therefore, we can apply the inverse of the Kolmogorov–Riesz theorem [25, Exerc. 4.34] to conclude that *I*_21_ converges to zero as *τ* → 0 uniformly in *m*. The analysis of *I*_22_ is more involved. We split the integral in several parts:

$$\begin{array}{ll} I_{22} & =\int_{0}^{T-\tau }\int_{\omega }y_{m}{\left(x,t\right)}^{2}z_{m}\left(x,t\right)\left(z_{m}\left(x,t\right)-z_{m}(x,t+\tau )\right)\text{dxdt}\\ & \;+\int_{0}^{T-\tau }\int_{\omega }y_{m}{\left(x,t+\tau \right)}^{2}z_{m}\left(x,t+\tau \right)\left(z_{m}\left(x,t+\tau \right)-z_{m}(x,t)\right)\text{dxdt}\\ & \;+\int_{0}^{T-\tau }\int_{\omega }\left(y_{m}{\left(x,t\right)}^{2}-y_{m}(x,t+\tau ){\;}^{2}\right)z_{m}\left(x,t+\tau \right)\left(z_{m}\left(x,t+\tau \right)-z_{m}(x,t)\right)\text{dxdt}\\ & ≕J_{1}+J_{2}+J_{3}. \end{array}$$

The first two integrals *J*_1_ and *J*_2_ are treated similarly as in Brenner and Masson [27, Lem. 3.11]. Indeed, let ⌈*s*⌉ denote the smallest integer larger or equal to *s*. Defining *n*_*m*_(*t*) := ⌈*t*/△*t*_*m*_⌉, we can formulate

$$z_{m}\left(x,t+\tau \right)-z_{m}\left(x,t\right)=\sum_{k=n_{m}\left(t\right)+1}^{n_{m}\left(t+\tau \right)}\left(z_{m,K}^{k}-z_{m,K}^{k-1}\right)$$

for *x* ∈ *K*, 0 ≤ *t* ≤ *T* − *τ*. With this formulation, we can bound *J*_1_, using the duality of ${\left\|\cdot \right\|}_{1,{𝒯}_{m}}$ and ${\left\|\cdot \right\|}_{-1,{𝒯}_{m}}$:

$$\begin{array}{ll} J_{1} & \leq \int_{0}^{T-\tau }\left(\sum_{K\in {𝒯}_{m}}m\left(K\right){\left(y_{m,K}^{n_{m}\left(t\right)}\right)}^{2}z_{m,K}^{n_{m}\left(t\right)}\sum_{k=n_{m}\left(t\right)+1}^{n_{m}\left(t+\tau \right)}\left(z_{m,K}^{k-1}-z_{m,K}^{k}\right)\right)\mathrm{dt}\\ & \leq \int_{0}^{T-\tau }\left(\sum_{k=n_{m}\left(t\right)+1}^{n_{m}\left(t+\tau \right)}{\left\|{\left(y_{m}^{n_{m}\left(t\right)}\right)}^{2}z_{m}^{n_{m}\left(t\right)}\right\|}_{1,{𝒯}_{m}}{\left\|z_{m}^{k}-z_{m}^{k-1}\right\|}_{-1,{𝒯}_{m}}\right)\mathrm{dt}\\ & \leq \frac{1}{2}\int_{0}^{T-\tau }\sum_{k=n_{m}\left(t\right)+1}^{n_{m}\left(t+\tau \right)}△t_{m}{\left\|{\left(y_{m}^{n_{m}\left(t\right)}\right)}^{2}z_{m}^{n_{m}\left(t\right)}\right\|}_{1,{𝒯}_{m}}^{2}\mathrm{dt}\\ & +\frac{1}{2}\int_{0}^{T-\tau }\sum_{k=n_{m}\left(t\right)+1}^{n_{m}\left(t+\tau \right)}\frac{1}{△t_{m}}{\left\|z_{m}^{k}-z_{m}^{k-1}\right\|}_{-1,{𝒯}_{m}}^{2}\mathrm{dt}\\ & \leq \frac{\tau }{2}\sum_{k=1}^{N_{m}}△t_{m}{\left\|{\left(y_{m}^{k}\right)}^{2}z_{m}^{k}\right\|}_{1,{𝒯}_{m}}^{2}+\frac{\tau }{2}\sum_{k=1}^{N_{m}}\frac{1}{△t_{m}}{\left\|z_{m}^{k}-z_{m}^{k-1}\right\|}_{-1,{𝒯}_{m}}^{2}, \end{array}$$

where the last inequality follows from [28, Lems. 4.1 and 4.2]. Let us remark that, for all *σ* = *K* ∣ *L* ∈ ℰ_int_, we can rewrite

$${\left(y_{K}\right)}^{2}z_{K}-{\left(y_{L}\right)}^{2}z_{L}=\frac{y_{K}+y_{L}}{2}\left(y_{K}z_{K}-y_{L}z_{L}\right)+\frac{y_{K}z_{K}+y_{L}z_{L}}{2}\left(y_{K}-y_{L}\right).$$

Then,

$${\left\|{\left(y_{m}^{k}\right)}^{2}z_{m}^{k}\right\|}_{1,{𝒯}_{m}}^{2}\leq m\left(\Omega \right){\left\|{\left(y_{m}^{k}\right)}^{2}z_{m}^{k}\right\|}_{L^{\infty }\left(\Omega \right)}^{2}+{\left\|y_{m}^{k}\right\|}_{L^{\infty }\left(\Omega \right)}{\left\|y_{m}^{k}z_{m}^{k}\right\|}_{1,{𝒯}_{m}}+{\left\|y_{m}^{k}z_{m}^{k}\right\|}_{L^{\infty }\left(\Omega \right)}{\left\|y_{m}^{k}\right\|}_{1,{𝒯}_{m}}.$$

Hence, *J*_1_ ≤ *Cτ* for some *C* > 0. An analogous estimation leads to *J*_2_ ≤ *Cτ*. It remains to estimate the integral *J*_3_. For this, we use, similar to the treatment of *I*_21_, the *L*^∞^ bounds on *y*_*m*_ and *z*_*m*_:

$$J_{3}\leq C\int_{0}^{T-\tau }\int_{\omega }\left|y_{m}\left(x,t+\tau \right)-y_{m}(x,t)\right|\text{dxdt}.$$

This expression converges to zero uniformly in *m* because of the relative compactness of (*y*_*m*_) in *L* ^2^(Ω_*T*_). We deduce from the previous computations that (48) holds true. Therefore, the product (*y*_*m*_ *z*_*m*_) converges strongly in *L* ^2^(Ω_*T*_), up to some subsequence, and in view of the convergences *y*_*m*_ → *y* strongly in *L* ^2^(Ω_*T*_) and *z*_*m*_⇀^*^*z* weakly* in *L*^∞^(0, *T*; *L*^∞^(Ω)), the limit of (*y*_*m*_ *z*_*m*_) equals *yz*, which finishes the proof.
